# Application of deep learning in cancer epigenetics through DNA methylation analysis

**DOI:** 10.1093/bib/bbad411

**Published:** 2023-11-20

**Authors:** Maryam Yassi, Aniruddha Chatterjee, Matthew Parry

**Affiliations:** Department of Mathematics and Statistics, University of Otago, Dunedin, New Zealand; Department of Pathology, Dunedin School of Medicine, University of Otago, Dunedin, New Zealand; Department of Pathology, Dunedin School of Medicine, University of Otago, Dunedin, New Zealand; Honorary Professor, UPES University, Dehradun, India; Department of Mathematics and Statistics, University of Otago, Dunedin, New Zealand; Te Pūnaha Matatini Centre of Research Excellence, University of Auckland, Auckland, New Zealand

**Keywords:** cancer epigenetics, DNA methylation, deep learning, systematic review

## Abstract

DNA methylation is a fundamental epigenetic modification involved in various biological processes and diseases. Analysis of DNA methylation data at a genome-wide and high-throughput level can provide insights into diseases influenced by epigenetics, such as cancer. Recent technological advances have led to the development of high-throughput approaches, such as genome-scale profiling, that allow for computational analysis of epigenetics. Deep learning (DL) methods are essential in facilitating computational studies in epigenetics for DNA methylation analysis. In this systematic review, we assessed the various applications of DL applied to DNA methylation data or multi-omics data to discover cancer biomarkers, perform classification, imputation and survival analysis. The review first introduces state-of-the-art DL architectures and highlights their usefulness in addressing challenges related to cancer epigenetics. Finally, the review discusses potential limitations and future research directions in this field.

## INTRODUCTION

Genetic and epigenetic alterations in DNA contribute to modify gene expression (GE) and are heavily implicated in human diseases, particularly in cancer [1]. Epigenetic alterations are defined as dynamic and heritable modifications to the genome that occur independently of a change in DNA sequence. Epigenetic alterations include DNA methylation, RNA-centered mechanisms (including noncoding RNAs and microRNAs), histone modifications and chromatin conformation, and are considered central molecular mechanisms in carcinogenesis/cancer progression [2]. DNA methylation is a stable epigenetic change that is particularly implicated in cancer development and metastasis [3, 4]. Consequently, epigenetic alterations in DNA play a significant role in clinical oncology, specifically as biomarkers and as targets for epigenetic drug therapies [5].

Changes in DNA methylation, either hypo- or hypermethylation, play an important role in carcinogenesis. DNA hypomethylation, the decrease in global DNA methylation, is recognized as a key feature of cancers [1, 6, 7]. Moreover, DNA hypermethylation, typically induced by the DNA methyltransferases family (abbreviations used in this paper are summarized in Supplementary Table 1 available online at http://bib.oxfordjournals.org/) [8], is also a common feature at pivotal genomic loci in cancer. DNA methylation changes contribute to the development of cancer [9] in a number of ways: DNA methylation alters signaling pathways that affect cellular processes such as cell cycle, DNA repair, cell growth and proliferation. Dysregulation of DNA methylation can also lead to inappropriate silencing of tumor suppressors or to the expression of oncogenes, and, as such, is an important example of an epigenetic driver of cancer growth [10–12] . Already, cancer-specific DNA methylation patterns are used in the diagnosis and treatment selection of cancer by detecting differential DNA methylation patterns as cancer biomarkers in biopsy specimens and blood samples [13] and there is a growing interest in developing commercialized tests that can diagnose colorectal cancer (CRC) in blood plasma based on DNA methylation [14].

The DNA methylation profile across the genome is vital to understand the influence of epigenetic mechanisms and modifications in cancer. Genome-wide epigenetic pattern of DNA methylation is explored and evaluated through developed platforms based on array and sequencing [15]. DNA methylation could be mapped by the whole-genome bisulfite sequencing (WGBS), which is currently the state-of-the-art technology for obtaining a comprehensive, nucleotide-resolution view of the methylome. While WGBS is comprehensive, it is relatively expensive and produces large volumes of data. For more targeted measurement or DNA methylation, the most commonly used approaches are Illumina Infinium HumanMethylation BeadChip arrays (e.g. 450 K and 850 K arrays), Enzyme–seq, reduced representation bisulfite sequencing (RRBS) and methylated DNA immunoprecipitation coupled with next-generation sequencing (MeDIP-seq). However, as these only cover a small proportion of the total CpG sites in the human genome, the scope of subsequent DNA methylation analyses can be considerably reduced [16, 17].

**Figure 1:**
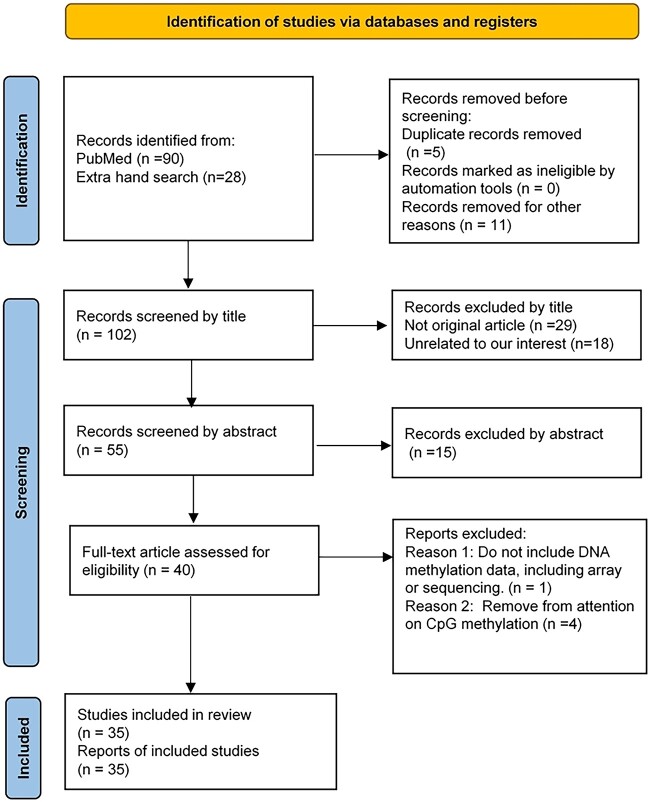
Overview of ML and DL applications in predication of cancer epigenetics.

Detecting cancer-specific DNA methylation patterns as unique signatures [18] and capturing the nonlinear interaction between the environment and epigenetic modifications, including aberrant DNA methylation in various cancers, currently pose important challenges. Furthermore, DNA methylation levels can also demonstrate widespread interindividual variation in normal individuals [19, 20]. Some of the challenges of analysis include dealing with large-scale and complex datasets, low signal-to-noise ratio and missing values in DNA methylation data. Consequently, there is a growing and recognized need for the application of advanced statistical and machine learning (ML) methods and DL technologies (as shown in Figure 1). Multimodal learning, multitask learning, feature selection and semi-supervised learning are critical features of applying ML and DL methods to DNA methylation data or multi-omics databases [21].

ML, developed historically as a subset of artificial intelligence (AI), has played a significant role in the development of autonomous cancer and disease diagnosis. The goal in ML is to develop models based on statistical associations and mathematical rules among features from a given dataset. ML algorithms are generally categorized into supervised learning and unsupervised learning [22]. In supervised methods, an outcome of interest or target is specified, such as a label, and a model is developed to predict the outcome from data. Some of the frequently employed algorithms in this ML category are support vector machine (SVM), random forest algorithms (RF), linear regression (LR) and logistic regression, including penalized variants such as least absolute shrinkage and selection operator regression (LASSO).

In unsupervised methods, no target is given and the algorithm tries to find natural clustering of similar data points. In this context, ML methods are specifically designed to discover hidden patterns or data groupings with little or no human intervention. Methods used include *K*-means clustering and hierarchical clustering, principal component analysis (PCA) and variants as well as dimensionality reduction techniques.

DL is a further specialized subset of ML that works in the same way as ML, but with vastly enhanced capabilities and approaches. DL algorithms can be regarded as the complex evolution of ML algorithms. DL models typically use a network of interacting layers to learn and discover information in data [23]. The intermediary layers can be understood as summarizing or representing the information that is flowing through the network. As well as posing serious computational challenges, DL also requires serious thought about the construction of the network, often called the DL architecture (see DL Architectures section). However, in both ML and DL approaches, the main aim remains: to create a model with minimal assumptions on the data generating process that will be generalizable to data not yet seen.

**Figure 2:**
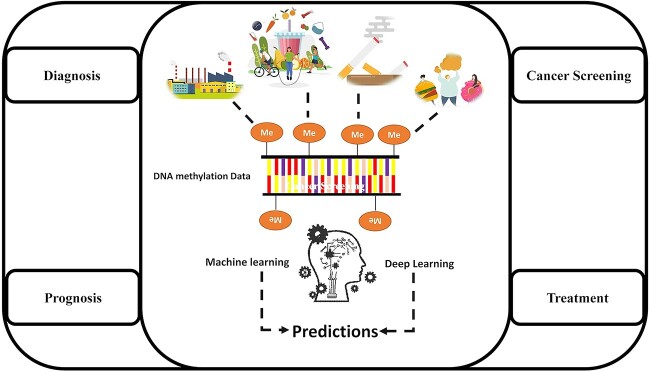
PRISMA flow diagram showing study selection process.

The focus of this systematic review is to investigate DL applications that address the challenges in cancer epigenetics by leveraging multi-omics datasets, specifically those involving DNA methylation data, and DL applications that analyze unimodal data obtained from both methylation array and sequencing platforms. The review was conducted using MEDLINE as the sole online database to find studies published until 23 December 2022. The following search terms consisted of a combination of DL-related keywords, DNA methylation and cancer including: ‘deep learning’, ‘artificial neural network’, ‘convolutional neural network’, ‘deep autoencoder’, ‘recurrent neural network’, ‘long-short-term-memory’, ‘gate recurrent unit’, ‘capsule neural network’, ‘transformer’, ‘cancer’ and ‘DNA methylation’. After an initial screening process, 35 studies were selected for further analysis from a total of 118 studies identified. For literature screening, a PRISMA flow diagram was prepared that displayed the number of studies included and excluded at each stage of the review, which is illustrated in Figure 2. These selected studies employed diverse DL architectures and computational methods to explore differentially methylated regions (DMRs) or CPG sites as cancer biomarkers, classify biopsy specimens and blood samples between case and control groups in various types of cancer from TCGA dataset or other data source, impute and predict CpG methylation states and estimate patient survival and survival-sensitive subtypes in cancer. The systematic review provides a comprehensive overview of recent advancements in DL-based approaches for cancer epigenetics research.

## DL ARCHITECTURES

With the significant improvement of computational power and the advancement of big data in recent years, DL has become one of the most successful approaches for dealing with the explosion of next-generation sequencing genomic data [24]. However, the architecture of a DL algorithm depends crucially on the task at hand and the type of data it will be deployed on. In this section, we describe some of the more important and successful DL architectures. For further in-depth information on DL architectures, please refer to the Supplementary materials, available online at http://bib.oxfordjournals.org/, of this article in DL Architecture which we have provided as a resource.

### Artificial neural network

**Figure 3:**
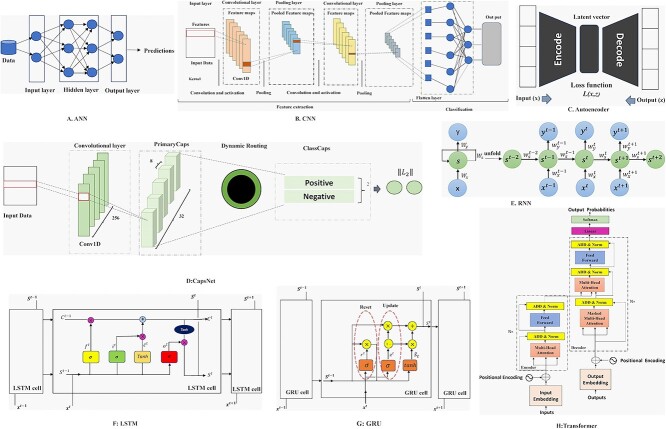
Architecture of the main DL models.

The design of artificial neural networks (ANNs) was inspired by the structure of the human brain: information is transmitted through layers of weighted, interconnected computational neurons [25]. ANNs typically have three layers: input, hidden (multiple) and output (Figure 3A). The hidden layer plays a crucial role in extracting important patterns, improving network efficiency by eliminating redundant information. The Multilayer Perceptron (MLP), a widely used ANN, computes the weighted sum of inputs from the previous layer. Each node’s output is determined by multiplying the output values of connected nodes in the preceding layer by their corresponding weights. The summed values undergo a nonlinear activation function to generate the node’s final output. ANNs are powerful and versatile models capable of learning any mathematical function given sufficient training and data.

### Convolutional neural network

Convolutional neural network (CNN) [26] is a DL model known for its outstanding performance in computer vision applications. It focuses on extracting local features from the data. CNNs consist of three main types of layers: convolutional layer, pooling layer and fully connected layer (Figure 3B). Convolution involves applying a kernel to the input data to filter and generate a feature map. Pooling reduces memory requirements and introduces spatial invariance—though this is a weakness in some contexts. Different types of pooling, such as max pooling and average pooling, can be used. The fully connected layer performs classification based on the extracted features, typically using the ReLU activation function in convolutional and pooling layers and the softmax function in the fully connected layer for appropriate classification.

### Deep autoencoder

Autoencoder (AE) [27] is an unsupervised ANN that is designed to learn efficient data encoding and representation to reconstruct the original input data. AE consists of two parts (Figure 3C): an encoder and a decoder. The encoder is used to generate a reduced feature representation from an initial input $x$ by a hidden layer *h*. The decoder is used to reconstruct the initial input $z$ from the encoder's output by minimizing the loss function $l\left(x,z\right)$. The AE converts high-dimensional data to low-dimensional data. Therefore, the AE is especially useful in noise removal, feature extraction, compression and similar tasks. There are four main types of AE: Denoising Autoencoder (DAE) [28], the Sparse Autoencoder [29], Contractive Autoencoder (CAE) [30] and Variational Autoencoder (CAE) [31].

### Capsule neural network

Capsule neural networks (CapsNets) were introduced [32] as an alternative to CNN. They aim to overcome the limitations of CNNs, such as the loss of valuable spatial information during max pooling. CapsNets address the challenge of recognizing new viewpoints by using capsules, which are groups of neurons representing specific entities or object parts. The CapsNet architecture consists of three layers: the conventional layer, primaryCaps layer and classCaps layer (Figure 3D). Each layer performs specific operations, such as converting input data into higher level features and conducting classification based on the length of capsules. Capsules in lower layers route their output to appropriate capsules in higher layers using a dynamic routing algorithm that considers orientation and magnitude similarities.

### Recurrent neural network

Recurrent Neural Networks (RNNs) possess internal memory, enabling them to capture and remember important information from previous inputs. This memory allows RNNs to make precise predictions for sequential data, such as time series, speech, text and DNA sequences [33, 34]. Unlike feed-forward networks, RNNs have a feedback loop connecting the previous output to the current step, enabling the influence of past steps on the current outcome (Figure 3E). Bidirectional RNNs learn information in both forward and backward directions simultaneously, providing bidirectional information flow for enhanced modeling of past and future contexts.

### Long short-term memory unit

Long Short-Term Memory (LSTM) [35] is an advanced type of RNN that addresses the issues of vanishing and exploding gradients. It introduces gates (input, output and forget) in its cell structure to capture both long- and short-term memory across time steps (Figure 3F). The input gate combines input and updates the state vector, the output gate combines current and previous state vectors, and the forget gate prevents updates to the current state. Bidirectional LSTM (BLSTM) extends LSTM by adding a reversed LSTM layer, allowing the model to utilize information from both directions of the input sequence. The outputs from both LSTM layers are combined for further processing.

### Gate recurrent unit

The Gated Recurrent Unit (GRU) [36] is a type of RNN that offers advantages over LSTM in terms of memory usage and speed. GRU has two gates (reset and update) that modulate information flow without a separate memory cell (Figure 3G). The update gate determines what information to keep or discard, while the reset gate controls the amount of past information to forget. The Bidirectional Gated Recurrent Unit (BGRU) combines two GRUs, one moving forward and the other backward, allowing information from both past and future to influence the current state. BGRU concatenates the states of the forward and backward GRUs.

### Transformer

Transformer-based models [37] have achieved impressive results in tasks such as machine translation, question answering and text classification. As shown in Figure 3H, they consist of an encoder and a decoder, employing attention-based architecture. The encoder includes sublayers for multihead self-attention and feed-forward connections, with skip connections and layer-wise normalization aiding training. The key innovation is the multi-head self-attention layer, allowing contextual understanding by associating relevant words in a context. Token embedding and position encoding are used to represent linguistic tokens numerically, and the decoder generates the output sequence using masked self-attention and encoder–ecoder attention layers. The final decoder output is passed through softmax to select the next word in the sequence. The detailed descriptions of all DL architectures and mathematical functions can be found in the Supplementary materials available online at http://bib.oxfordjournals.org/.

## APPLICATION OF DL TECHNIQUES TO ANALYSE THE MULTI-OMICS DATA AND DNA METHYLATION DATA IN EPIGENETIC CANCER

In this study, we identified a total of 35 research articles that utilized various DL architectures. The articles were categorized into four groups: (1) diagnosis or classification of cancer epigenetics and diseases, (2) prediction of CpG methylation states and imputation of missing values, (3) biomarker discovery in tissue and cfDNA samples and (4) estimation of patient survival and survival-sensitive subtypes in cancer. The findings of these studies are summarized in Tables 1–4, respectively, which includes details such as the pipeline name, DL framework, DL architectures used, the specific cancer types studied and the research focus in terms of multi-omics datasets and unimodal data (DNA methylation). Overview of the performance metrics employed in each category is provided (Supplementary Tables 2–5 available online at http://bib.oxfordjournals.org/) along with the calculation and definition of these metrics (Supplementary Tables 6 available online at http://bib.oxfordjournals.org/).

**Table 1:** List of DL methodologies in cancer epigenetics subtype classification

Ref.	Pipeline name/ Software link	Framework	DL Architecture	Disease(s)	Omics data	Research direction
Multi-omics datasets						
[38]	MetaCancer https://github.com/SomayahAlbaradei/MetaCancer	Keras and TensorFlow	CVAE, FC layer	11 types of cancer from TCGA projects	RNA-Seq microRNA-Seq, DNA methylation	The overarching goal would be to accurately classify tumor samples as primary or metastasized, with the target of informing treatment decisions and improving clinical outcomes for cancer patients.
[39]	https://drive.google.com/open?id=1LsYe3ypiweox2OSmnD5LDaYMJePeozDd	NA	FNNs	Uterine cervical cancer	DNA methylation, GE	The objective of this investigation is to predict GE values based on prefiltered DNA methylation data and subsequently identify DEGs which can be utilized to classify the cervical cancer label.
[40]	CapsNetMMD https://github.com/ustcpc/CapsNetMMD	Keras and TensorFlow	CapsNet	Breast Caner	mRNA expression, DNA methylation, Two forms of DNA CNAs	Leveraging capsule networks to integrate multi-omics data has the potential to yield more precise and comprehensive analyses for the identification of breast cancer-related genes and the elucidation of cancer progression within complex biological systems.
[41]	DDAE-MLP https://github.com/vd4mmind/multiOmicsIntegration	Keras and TensorFlow	DAE MLP	LIHC	DNA methylation, RNA-seq, CNV	The objective of this research would be to estimate GE by investigating the intricate relationships between genomics CNV and epigenomics DNAm information at a higher dimensional level, thereby contributing to the advancement of our understanding of the regulation of GE.
[42]	DFNForest https://github.com/tuiainao316/datasets	NA	Stacked AEs, Deep flexible neural forest (DFNForest)	Brest invasive carcinoma (BRICA) Glioblastoma multiform (GBM) Ovarian cancer (OV)	GE, DNA methylation, miRNA expression	The objective would be to utilize the new HI-DFNForest method to integrate multi-omics data from TCGA and accurately classify distinct subtypes of cancer, with the potential to enhance our understanding of the underlying mechanisms of these diseases and inform more personalized treatment approaches.
[43]	GCBMI	Keras and TensorFlow	DNN	Gastric cancer	DNA methylation, GE	The goal of this research would be to employ a DL-based classifier model to identify potential biomarkers for gastric cancer, with the potential to enhance our understanding of the underlying molecular mechanisms of the disease and inform more precise diagnostic and therapeutic strategies.
Unimodal data (DNA methylation)						
[44]	NA	NA	MLP	7 types of cancer	DNA methylation	The objective would be to utilize large-scale DNA methylation data to accurately classify different types of cancer diseases, with the aim of advancing the understanding and treatment of these conditions
[45].	https://github.com/rahulgomes19/Deep_Learning_Methylatin	Keras and TensorFlow	Sequential DNN	Breast cancer	DNA methylation	The aim of the research would be to predict breast cancer patient outcomes and identify the specific genes that significantly contribute to this prediction, utilizing advanced analytical methods and techniques.
[46]	NA	NA	DNN	Malformations of cortical development (MCD)	DNA methylation	The objective would be to utilize DNA methylation profiles from formalin-fixed and paraffin-embedded (FFPE) tissue obtained during epilepsy surgery to diagnose malformations of cortical development disease, with the potential to improve clinical decision-making and patient outcomes.
[47]	MethylCapsNet and MethylSPWNet https://github.com/Christensen-Lab-Dartmouth/MethylCapsNet	Pytorch	CapsNet, MLP, GLRM in DNN	CNS	DNA methylation	The goal of the study would be to accurately classify patients with CNS tumors into 38 distinct tumor types, utilizing DNA methylation analysis as a basis for classification.
[48]	MethylNet https://github.com/Christensen-Lab-Dartmouth/MethylNet	Pytorch	VAE	32 different cancer subtypes, Breast cancer	Six public DNA methylation data sets for a range of various tasks: Age, Cell type, 32 different cancer Subtypes, Current smoker and never smoker, Breast tumor subtyping	The objective would be to employ predictive modeling techniques to simultaneously tackle multiple tasks, including cell-type deconvolution, pan-cancer subtype classification, age regression, and smoking status classification.
[49]	DISMIR https://github.com/XWangLabTHU/DISMIR	Keras and TensorFlow	CNN, BLSTM	HCC	cell-free DNA methylation data	The aim of the study would be to diagnose hepatocellular carcinoma (HCC) utilizing whole genome bisulfite sequencing of circulating cell-free DNA (cfDNA) at low sequencing depth, with the potential to improve diagnostic accuracy and facilitate earlier detection of the disease.
[50]	iCancer-Pred https://github.com/Huerhu/iCancer-Pred	Keras and TensorFlow	FC layer	7 different cancer subtypes from TCGA	DNA methylation	The objective would be to identify patients with cancer across seven distinct subtypes and accurately classify the specific subtype of cancer present, utilizing advanced analytical techniques to inform more precise diagnosis and treatment strategies.
[51]	NA	Keras and TensorFlow	VAE	LUAD LUSC	DNA methylation	The aim of this research would be to employ VAEs on epigenetic data from patients with lung cancer in order to distinguish between the original subtypes of the disease, thereby enabling more targeted and effective treatment strategies.
[52]	Cancer origin prediction https://github.com/thunder001/Cancer_origin_prediction	Keras and TensorFlow	MLP	18 different cancer origins from TCGA	DNA methylation, clinical patient information	The aim of this study would be to leverage a DL-based classifier methodology to accurately predict the origins of cancer using datasets from TCGA, potentially contributing to the advancement of precision medicine in oncology.

**Table 2:** List of DL methodologies in missing value imputation and prediction of CpG methylation data

Ref.	Pipeline name/ Software link	Framework	DL Architecture	Disease(s)	Omics data	Research direction
Multi-omics datasets						
[66]	https://github.com/gevaertlab/BetaVAEImputation	Keras and TensorFlow	VAE	RNA-seq, DNA methylation	RNA-Seq, DNA methylation	This goal of this study would be to develop a VAE-based DL imputation framework for DNA methylome and transcriptome data.
Unimodal data (DNA methylation)						
[67]	DeepCpG https://github.com/PMBio/deepcpg, doi: 10.5281/zenodo.322423	Keras and TensorFlow	BiGRUs CNNs FC layers	HCC	scBS-seq, and scRRBS-seq on 5 different datasets: Serum, 2i, HCC, HepG2, mESC	The aim of this research study would be to utilize WGBS and RRBS data to accurately predict the methylation states of individual cells in patients with HCC.
[68]	BiLSTM-5Mc https://github.com/taigangliu/BiLSTM-5mC	Pytorch	BLSTMs FC layers	NA	Cell lines of the SCLC database	The objective of this research would be to recognize 5-methylcytosine (5mC) sites within a genome-wide database of DNA promoters, with the potential to enhance our understanding of the epigenetic regulation of GE.
[69]	CPG transformer https://github.com/gdewael/cpg-transformer	Pytorch	Transformer CNNs	HCC Human monoclonal B-cell lymphocytes (MBL)	scBS-seq, and scRRBS-seq on 5 different datasets: Ser, 2i, HCC, MBL, Hemato	The goal of this research study would be to utilize WGBS and RRBS data to accurately impute and denoise single-cell methylation data in patients with HCC and BML.
[73]	D-GPM	NA	FC layers	Different cancer types from TCGA	DNA methylation	This study, introduces a novel approach to prognosticate the overall level of promoter methylation in the genome predicated upon the promoter methylation patterns of the landmark genes from TCGA.
[70]	MRCNN https://github.com/TQBio/MRCNN	Keras and TensorFlow	CNNs	3 types of cancers: Brain, Colon, Lung	DNA methylation	In this study, a computational approach that relies on CNNs is proposed for the purpose of predicting the methylation status of DNA throughout the entire genome at the resolution of CpG sites
[71]	INTERACT https://github.com/LieberInstitute/INTERACT	Keras and TensorFlow	CNNs, Transformer, FC layers	Fine-tuning: four different tissue: Brain, Blood, saliva, Buccla Pre-training: Hippocampus	DNA methylation	The aim of this study would be to estimate DNA methylation level of CpG sites from local DNA sequencing by developing a DL model that integrates CNNs and Transformer

**Table 3:** List of DL methodologies in identification of epigenetic biomarkers for cancer diagnosis and prognosis

Ref.	Pipeline name/ Software link	Framework	DL Architecture	Disease(s)	Omics data	Research direction
Unimodal data (DNA methylation)						
[53]	NA	NA	ANNs	Ovarian cancer	cell-free DNA methylation data	The objective of this research study would be to discovery remarkable CpG markers as a noninvasive marker in Ovarian cancer patients.
[53]	NA	NA	ANNs	Lung cancer	cell-free DNA methylation data	The goal of this research would be to identify CpG markers located within 4389 CpGs in coding genes and 1812 CpGs in noncoding DNA regions, potentially contributing to our understanding of epigenetic regulation of GE and aiding in the development of more precise diagnostic and therapeutic strategies.
[54]	Cancer methylation https://github.com/BiaoLiu2017/Cancer-methylation	Keras and TensorFlow	multi-layer FNNs	27 cancer types from TCGA	DNA methylation, cell-free DNA methylation data	The aim of this study would be to recognize the two categories of DNA methylation markers, CpG markers and promoter markers, to diagnose diverse cancer types based on tissue and liquid biopsy.

**Table 4:** List of DL methodologies in estimating patient survival and survival-sensitive subtypes in cancer

Ref.	Pipeline name/ Software link	Framework	DL Architecture	Disease(s)	Omics data	Research direction
Multi-omics datasets						
[55]	ML_ordCOX https://github.com/bhioswego/ML_ordCOX.	R and Python	BLSTMs FC layers	Breast cancer	mRNA-seq, DNA methylation	The main approach of this study would be to make prognostic assessments in the context of survival outcomes involves the utilization of GE and DNA methylation characteristics as predictive features.
[56]	SdA https://github.com/tjgu/cancer_subtyping.	Keras and TensorFlow	Stacked denoising AEs	Kidney Renal Clear Cell Carcinoma (KIRC)	GE, Protein expression, miRNA-seq, DNA methylation, CAN	The goal of this research study would be to classify KIRC patients to two survival-sensitive groups based on integrating five genomics datasets.
[57]	NA	NA	AEs	Lung cancer	mRNA-seq, miRNA, DNA methylation, CNVs	In this study, stratify the survival risk for Lung cancer adenocarcinoma patients based on DL autoencoding model by omics data.
[58]	Multi-view Factorization Autoencoder (MAE) https://github.com/BeautyOfWeb/Multiview*AutoEncoder*	Pytorch	Multiple AEs	Bladder urothelial carcinoma Brain lower grade glioma	DNA methylation, miRNA sequencing, protein expression, clinical data information	The objective of this research study would be to integrate multi-omics datasets and biological domain knowledge such as molecular interaction using Multi-view Factorization AEs to predict clinical outcomes.
[59]	i-Modern	NA	AE, FC layers	Glioma	RNA-seq, miRNA-seq, CNVs, DNA methylation, protein expression, DNA-seq (somatic mutations), clinical patient information	The objective of this study would be to develop an integrated Multi-omics DL network method for gliomas that has the capability to accurately classify patients based on their prognosis.
[60]	NA	Keras and TensorFlow	AE	CRC	DNA methylation RNA-seq miRNA-seq	In this research study, build an AE-based model to estimate the prognosis of CRC by integrating multi-omics datasets from the TCGA for predicting the prognostic factors for CRC.
[61]	NA	Keras and TensorFlow	AEs	NSCLC which consists of three histological subtypes: LUAD, LUSC, Large cell carcinoma	mRNA-seq, miRNA-seq, CNVs, DNA methylation, RPPA (reverse phase protein array), DNA-seq (somatic mutations), clinical patient information	The aim of this study would be to predict survival-associated subtypes in NSCLC like LUDA and LUSC using the multi-omics datasets from six categories from TCGA
[62]	AE *GitHub*—*Deep learning-omics/autoencoder*	Keras and TensorFlow	AE	Glimoas tumors	RNA-seq, DNA methylation	In this study, provide an AE-based approach was used to recognize two survival-sensitive subtypes of Glimoas by integrating RNA seq and DNA methylation and supporting personalized treatment strategies.
[63]	Breast Cancer Survival Integration https://github.com/tongli1210/BreastCancerSurvivalIntegration	Keras and TensorFlow	AEs	Breast cancer	GE, DNA methylation, miRNA expression, CNVs	The main approach of this study would be to improve breast cancer OS prediction by integrating multi-omics data
Unimodal data (DNA methylation)						
[64]	Deep Survival Epigenome-Wide Association Study (EWAS) https://github.com/LorenzoDominoni/DeepSurvEWAS	Pytorch	multi-layer FNN	Breast cancer	Blood DNA methylation	In this study, identify DNA methylation patterns in blood associated with the duration between the onset of a disease and its detection or diagnosis (Time to Diagnosis) that would enhance the reliability and biological significance of the result in a survival analysis scenario.

### Epigenetic cancer subtype classification

#### Multi-omics datasets

MetaCancer [38] is a DL approach for classifying primary and metastatic cancers using a combination of CVAE and DNN with FC layers. The study utilized RNA-Seq, microRNA-Seq and DNA methylation data from 11 different cancer types sourced from the TCGA project. During the preprocessing phase, the focus was on identifying and mapping specific CpG islands located 1500 base pairs away from the transcription start sites (TSSs) of genes. The methylation values of these islands were averaged using the Enhancer Linking by Methylation/Expression Relationship (ELMER) R package. Additionally, missing expression values were imputed among the same samples in three input databases.

The CVAE was employed to automatically extract features from the multi-omics datasets, while the DNN was trained in a supervised manner to discriminate between the two classes. The encoder and decoder parts of the variational autoencoder (VAE) architecture consisted of two convolutional layers and one dense layer (dense layer and two deconvolution layers, respectively). This architecture enabled the recognition of local patterns independent of their position in the data, facilitating effective feature extraction. Subsequently, the latent vector extracted by the CVAE was used as input to train the DNN for the accurate classification. This study introduced a novel methodology for integrating DNA methylation and GE data in uterine cervical cancers. The MetaCancer model achieved a high accuracy of 88.85%, with an AUC of 91.19%, precision of 91.65%, sensitivity (recall) of 87.69%, F1 score of 90.44% and specificity of 89.61%.

This approach utilized a multistep process, starting with the removal of outliers and LR to predict GE data from preprocessed methylation data, using TCGA GE data as a reference. The study then identified significantly differentially expressed genes (DEGs) with an FDR cut-off of less than 0.001, using Empirical Bayes test and Limma downstream analysis. Finally, the proposed approach employed DL techniques, specifically feed-forward neural networks (FNNs), to interpret the DEGs [39]. The methodology was evaluated with an AUC of 85, an average accuracy rate of 90.69%, an average sensitivity rate of 73.97%, an average specificity rate of 97.63% and an average precision rate of 93.38%.

The CapsNeTMMD method, developed by [40], aims to analyze multi-omics datasets in breast cancer, including mRNA expression, DNA methylation and two types of DNA copy-number alterations (CNAs). The method combines these datasets to create gene feature matrices and extracts relevant breast cancer genes from DisGeNET (https://www.disgenet.org/) for supervised classification. The feature matrix is normalized and reshaped, serving as input to a CapsNet classifier with two convolutional layers and one fully connected layer. The first layer operates on the reshaped matrix, while the second layer works on feature maps generated from the first layer. These feature maps are transformed into 32 channels of 8D capsules in PrimaryCap. To enhance the classifier 's learning ability, the method applies instantiation parameters and dynamic routing mechanism settings. The study achieved an AUC of 94.6%, a sensitivity rate of 88.7% and a specificity rate of 90%. This research provides a promising methodology for the classification of breast cancer patients using multi-omics datasets.

A DL approach called DDAE-MLP [41] was proposed to predict GE values and classify tumor and normal samples in Liver Hepatocellular Carcinoma (LIHC). The method comprises of several steps. Initially, a preprocessing stage is conducted, which involves calculating the average methylation value within a specific range from the TSS, identifying common samples for all three omics data, imputing missing data and normalizing the data using the Scikit-learn packages. Then, a DL regression model is constructed using a DDAE network for feature extraction and dimensionality reduction, combined with an MLP network for multi-output regression to predict GE values. Finally, the DL framework is utilized to extract features that enable the classification of tumor and normal samples, thus validating the proposed model. The authors achieved an impressive performance on the regression task with a negative logarithmic scale of RMSE = 1.33, *R*^2^ = 0.96 and a correlation coefficient of 0.68. Additionally, the proposed model achieved high accuracy 95.1%, precision 96%, recall 95% and F1-score 95% on the classification task.

The HI-DFNForest [42] methodology was developed to address the challenge of cancer subtype classification by integrating multi-omic data types. The efficacy of this method was evaluated on three TCGA cancer types (breast invasive carcinoma, glioblastoma multiform and ovarian cancer), by integrating GE, miRNA expression and DNA methylation data. Stacked AEs were employed to learn the representations of each omics data, and subsequently, all learned representations were integrated into a single AE to learn complex data patterns. The learned complex representations were then utilized as input to the deep flexible neural forest (DFNForest) model to classify cancer subtypes. Furthermore, the DFNForest model leverages the concept of ensemble learning, and thus, the diversity and accuracy of the model can be effectively enhanced. Moreover, the accuracy, sensitivity (recall), F1-score and precision of the classification results were also reported 74.32, 81.33, 88.66 and 80.66% among BRCA, GBM and OV. The F1-score and precision were also high, indicating that the model was able to achieve a good balance between precision and recall.

A novel GCBMI model [43] was applied to gastric cancer by integrating DNA methylation and GE data using DL techniques. The study consisted of three stages: data preprocessing, gene selection using mutual information, fold change and *t*-test methods, and the construction of a DL network as a classifier. The DL network had two input layers for GE and DNA methylation data, six hidden layers with the Relu activation function and 100 nodes each, and an output layer with one node using the Sigmoid activation function. The model aimed to identify DEGs and differentially methylated positions while avoiding redundancy in a new dataset created from tumor and normal samples. The GCBMI model achieved excellent performance with AUC 98%, accuracy 98%, precision 99%, sensitivity (recall) 98% and F1 score 99%.

#### Unimodal data (DNA methylation)

A two-stage DL approach was proposed [44] for classifying malignancy in DNA methylation data across seven different types of cancer from the TCGA project. In the first stage, the study used an unsupervised metaheuristic technique based on genetic algorithms to perform feature selection and reduce the dimensionality of the DNA methylation loci. In the second stage, the authors constructed two DL models, including a binary classification model to classify malignancy in samples and a multi-classifier model to classify pan-cancer statuses for selected cancer diseases based on malignancy information and common features. The binary and multi-class classification models consistently demonstrated outstanding performance, boasting a remarkable AUC of 90.92% and an impressive accuracy rate of 98.33%. For the various cancer types, the F1 score, precision and recall exhibited exceptional values 98.91, 98.77 and 98.32%, respectively. Furthermore, the binary classification model showcased a commendable MMC of 93.5%.

A three phase method was developed to classify breast cancer patients using methylation data. In the first phase, preprocessing steps were performed, including the removal of specific CpG sites and handling missing values using various methods such as zero, *k*-nearest neighbor, mean and iterative imputation. The synthetic minority oversampling technique was used to address class imbalance in the dataset. In the second phase, dimensionality reduction methods, namely ANOVA and RF, based on ML and statistical approaches, were applied to reduce the dataset's dimensionality. Finally, two variants of a sequential deep neural network (DNN) were implemented, considering the dataset size (27 K and 450 K), to classify breast cancer patients. The study achieved high accuracy 98.75% and precision 99.99% in classifying breast cancer patients. The AUC was 98.75%, indicating excellent performance. The sensitivity (recall) and F1 score were 97.6 and 98.73%, respectively [45].

An ML- and DP-based method was developed to classify MCD patients using methylation data. The study used classical ML algorithms, such as SVM, KNN, RF and tree classifier to classify MCD patients. Additionally, the study proposed a stacking ML algorithm, which combines the predictions of several ‘weak’ classifiers to improve prediction robustly and accurately. Furthermore, the study modeled a DL network consisting of three subsequent layers. To be consistent with plots of the disease clusters, the study transformed the predictions of the ML and DL models into a 2D space via Uniform Manifold Approximation and Projection dimensionality reduction. The DL network had an AUC of 100% and an accuracy of 94%. The precision and sensitivity were both 98% [46] .

MethylCapsNet and MethylSPWNet [47] are DL approaches that are applied to methylation data to predict categorical outcomes in the Central Nervous System (CNS). MethylCapsNet utilizes a capsule network (CapsNet) to group CpGs into biologically relevant capsules, considering gene promoter context, CpG island relationship and other annotation information. MLPs are then applied to each capsule to extract context-specific features, and the dynamic routing algorithm in the CapsNet model facilitates information flow between child and parent capsules to estimate disease or categorical outcomes. On the other hand, MethylSPWNet employs a group LASSO Regression model, where methylation values for CpGs within each gene are transformed into a single value using gene-specific CpG weight matrices. These matrices are updated through the training of MLPs to predict the expected outcome, and L1 regularization is applied to preserve important genes while excluding unnecessary ones. Both methods exhibited remarkable accuracy, both achieving an impressive 98%. Additionally, their precision, sensitivity (recall) and F1 score all reached 97%.

Methylnet, a pretrained VAE [48], was utilized to extract biologically meaningful features in the encoder part for multi-output regression tasks including age prediction and cell-type deconvolution, and classification tasks including pan-cancer subtypes and smoking prediction. The authors employed an autonomous hyperparameter scanning technique to optimize the model parameters and determined the contribution of important CpGs to each prediction using Shapley Feature Attribution method. The accuracy of Methylnet for pan-cancer classification was reported to be 97%, with precision, sensitivity (recall) and F1 score all at 97%, respectively. For age estimation by regression task, Methylnet achieved an *R*^2^ value of 0.96 and an MAR of 3.0 [65].

DISMIR was developed to determine the source of reads in plasma cfDNA WGBS data and estimating the proportion of tumor-derived reads. DISMIR comprises four main phases: identifying cancer-specific DMRs as candidate biomarkers, screening out reads located in those DMRs, building and training a CNN-Bidirectional LSTM model to predict a *d*-score value, and estimating the fraction of tumor-derived reads by maximizing the posterior probability of a plasma sample using all *d*-scores predicted by the DL-based model in the previous stage. In this study, DISMIR was applied to methylation information of cfDNA and DNA sequence data in HCC patients, achieving an impressive AUC 99.69%, a sensitivity (recall) of 93.94% and a specificity of 100%. Furthermore, the Pearson correlation between tumor size and the ratio of tumor-derived reads was found to be 0.88, with a *P*-value of 6.68 × 10^−5^ [49].

iCancer-pred [50] was employed as a diagnostic tool for the identification of multiple cancer types using DNA methylation data. The approach utilized a two-stage feature selection process to reduce dimensionality, wherein the coefficient of variation and elastic network techniques was applied in the first and second stages, respectively. Additionally, two fully connected neural networks were employed to perform binary and multiclass classification tasks. The binary classifier utilized one output node and a sigmoid activation function to distinguish between healthy and cancerous states, while the multiclass classifier employed eight output nodes and a softmax activation function to differentiate between healthy and seven different cancer subtypes. The iCancer-pred tool demonstrated high performance, with an AUC of 99.68%, an accuracy rate of 98.37%, an MCC of 92.95%, a sensitivity rate of 98.15% and a specificity rate of 99.53%.

A DL methodology utilizing VAE was developed to analyze DNA methylation datasets for lung adenocarcinoma (LUAD) and lung squamous cell carcinoma (LUSC) obtained from the TCGA. VAEs were employed to generate a biologically significant latent space by merging the datasets, accurately capturing the distribution of distinct sample subtypes. The high-dimensional features were encoded to a lower dimensional intermediate layer and then decoded back to the original dimensionality using a nonlinear combination. To gain insights into the resulting 100D space, t-Distributed Stochastic Neighbor Embedding was applied to reduce the dimensionality further to 2. Logistic regression classifiers were trained using the 2D features to evaluate the performance of the VAE-generated latent space in classifying among four groups (LUAD-01, LUAD-02, LUSC-01, LUSC-11). The study accomplished useful results, achieving an AUC and accuracy rate both reaching 91.50%, with a sensitivity rate of 78.75% and an F1 score of 81.25% [51].

A DL-based classifier model was developed to predict cancer origins using DNA methylation data. Statistical techniques such as one-way ANOVA and Tukey's HSD test were applied to reduce dimensionality and eliminate noise in the data. These techniques filtered CpG sites with insignificant differences across tissues and removed CpG sites with minimal variation in mean beta values. The resulting dataset included 10 360 CpG sites, which were used as input for an MLP model. The MLP model consisted of 2 hidden layers with 64 nodes each, using a Relu activation function and an output layer with 18 nodes, employing a softmax activation function. The results demonstrated a model with exceptionally high sensitivity and specificity, scoring 94.48 and 99.80%, respectively [52].

### Missing value imputation and prediction of CpG methylation data

#### Multi-omics datasets

The imputation of missing values in GE and DNA methylation data (DM) was explored using VAE methodology [66]. The study focused on imputing missing values in both Missing Completely at Random (MCAR) and Missing Not at Random (MNAR) scenarios. MNAR simulations were motivated by different real-world conditions specific to either GE data or DM testing data. The iterative imputation process involved replacing missing values with random values, computing the latent variable distribution, using the mean of the latent variable distribution as input for the decoder, computing the distribution of reconstructed data, using the mean of the reconstructed data distribution as the imputed values, replacing missing values with the imputed values, and keeping nonmissing values unchanged. The VAE method was compared against the performance of common ML models such as KNN and singular value decomposition (SVD) in MCAR cases of 5, 10 and 30% and different conditions of MNAR for GE and DM. The VAE method performed better than SVD and KNN for GE and DM datasets into two primary domains: MCAR and MNAR scenarios. Typically, the VAE's RMSE values for GE and DM data were 0.31 and 0.70, respectively.

#### Unimodal data (DNA methylation)

DeepCpG [67] is a computational model that was developed for predicting the binary methylation states of CpG sites in single-cell DNA methylation sequencing data. The model utilizes a DL approach, incorporating a CNN to extract features from the DNA sequence and a BGRU to recognize features from the CpG neighborhood across all cells. The prediction of CpG methylation is based on information derived from both local DNA sequence windows and observed neighboring methylation states obtained from read counts mapped to a reference genome. While the DNA module focuses on detecting motifs, the CpG module compresses patterns of CpG states into a feature vector. Additionally, a multitask Joint module combines the outputs of the previous modules to predict the binary CpG methylation states of target CpG sites for multiple cells using FC layers. The performance of DeepCpG was evaluated on scBS-seq and scRRBS-seq datasets from five different cell types: Serum, 2i, HCC, HepG2 and mESC in HHC patients. The results demonstrated that the model achieved high accuracy in predicting CpG methylation states with AUC values ranging from 82 to 97%, with an overall average AUC of 92.4%.

The BiLSTM-5Mc [68] is a method developed for identifying 5-methylcytosine (5mC) sites in nucleotide sequences. This approach utilizes both the one-hot encoding and nucleotide property and frequency (NPF) methods to represent the nucleotide sequences. To address the imbalance between positive and negative samples, the training dataset’s negative samples are randomly divided into multiple groups of equal size. One group is then combined with an equal number of positive samples to create a balanced training subset. The BiLSTM-5Mc approach employs two BLSTM models with FC layers to capture sequence-order and position-specific information from the one-hot and NPF features. Furthermore, the method employs a simple majority voting technique to determine whether a query sequence is a 5mC sample or not based on multiple predictive results from the DL model. The performance of BiLSTM-5Mc was evaluated on cell lines from the SCLC database. The results demonstrated high accuracy in predicting 5mC sites, with an AUC of 96.35%, accuracy of 93.03%, sensitivity of 86.61%, specificity of 93.74% and MMC of 63.84%.

The CpG transformer [69] is an architecture based on the Transformer neural network that was specifically designed to handle partially observed methylation matrices. It effectively processes the input data using axial attention and sliding window self-attention. The model takes the CpG matrix, genomic positions and DNA sequences surrounding the target CpG as inputs. It consists of two stages: in the first stage, CNNs with two convolutional layers and max-pooling layers are used to process the input data. In the second stage, transformer layers with axial attention are employed to model interactions between matrix entries. This includes separate self-attention operations to capture dependencies within rows and columns. Furthermore, the model leverages the autocorrelation between neighboring methylation sites, utilizing the correlation between closely located CpG sites on the genome. The performance of the CpG transformer was evaluated using scBS-seq and scRRBS-seq data from five different datasets such as Ser, 2i, HCC, MBL, Hemato from HCC and MBL patients. The results showed that the approach achieved high performance, with an overall average AUC of 91.68% across the different datasets.

A multilayer neural network called the D-GPM [73] was developed to handle large-scale multitask problems. The model utilized promoter methylation data obtained from the Illumina Human Methylation 450 k data from TCGA, specifically from 902 landmark genes and 21 645 target genes. The D-GPM model has 902 input units and 21 645 output units, and it calculates the average *β* value of all the probes located in the promoter regions of a certain gene to determine its corresponding promoter methylation level. The model applies this promoter methylation level to the landmark genes to predict the methylation profile of target. The D-GPM was compared against the performance of classical ML models, including RT, LR and SVM, by evaluating the mean absolute error (MAE) and Pearson correlation coefficient (PCC) criteria. The results demonstrated that D-GPM outperformed classical ML methods in terms of both MAE and PCC, achieving an MAE of 0.03 and a PCC of 0.82.

The MRCNN [70] is a specialized model that predicts CpG site methylation status across the entire genome. It was trained and tested independently using the WGBS dataset, where methylation levels were represented as ratios from 0 to 1 for each CpG. To establish associations between DNA sequence patterns and methylation levels, a 400 bps DNA sequence fragment centered at the methylation site was extracted. The DNA sequence was then converted into a 2D array (400 × 4) using one-hot encoding and used as input for CNNs. The CNN model consisted of an input layer, followed by a convolution layer with a kernel size of 1 × 4 and 1 × 6 feature maps. The output tensor was reshaped into a 20 × 20 tensor, which was further processed by traditional convolution and pooling layers using a 3 × 3 convolution kernel, max pooling and Relu activation function. Two additional convolution layers with the same size and step size as the second layer were included, without pooling or activation functions. The final layer was an FC layer. To evaluate the model prediction performance, two aspects were considered: regression errors and binary classification performance. The average regression errors for hypermethylation, hypomethylation and intermediate methylation status were reported as (RMSE, MAE) = (0.21, 0.19). The authors also assessed the robustness of the MRCNN model for more complicated methylation mechanisms from different types of tissues, including brain, lung and colon. On average, they reported the following performance metrics: AUC 93.2%, accuracy 89.9%, sensitivity 91.3% and specificity 85.3%.

The INTERACT model [71] is a recent development used to predict the effects of genetic variations on DNA methylation (DNAm) levels in human brain. It involves a pretraining step using a dataset of 26 million CpG sites in the hippocampus, measured with WGBS. This pretrained model is then fine-tuned using the EPIC array to predict DNAm levels in specific tissue samples. The model consists of a preprocessing stage and three main modules: CNN, a Transformer and an FC network. The preprocessing stage uses a one-hot encoded DNA sequence, with the CpG site located at the center of the DNA sequence. The CNNs module consisting of three convolution layers followed by max pooling and dropout layers. The output is then passed to the transformer module to capture long-range dependencies. Finally, the transformer’s learned features are processed by an FC network to predict DNAm levels at the relevant CpG sites. The performance of the INTERACT model was evaluated on four different tissues: brain, blood, buccal and saliva. The results showed that the Spearman correlation of 0.78, the root mean squared error of 0.15 and an AUC of 82.5%.

### Identification of epigenetic biomarkers for cancer diagnosis and prognosis

#### Unimodal data (DNA methylation)

To classify circulating cell-free DNA (cfDNA), methylation data in ovarian cancer were evaluated using two stages. The first stage involved selecting the top 2000 and 5000 CpGs in promoter regions using the Recursive Feature Elimination method and logistic regression for classification. In the second stage, six AI models were employed on all CpGs located in intragenic regions, including RF, SVM, ANN, Prediction Analysis for Microarrays (PAMs), Linear Discriminant Analysis (LDA) and Generalized Linear Models (GLMs). Finally, a set of CpG markers were proposed in two parts as binary classification in ovarian cancer. The performance of the AI models was evaluated using AUC, sensitivity and specificity. The DL model achieved an AUC of 100% with a sensitivity of 100% and specificity of 88% [53] .

In addition, a method for detecting lung cancer using cfDNA methylation analysis and AI was developed by Bahado-Singh et al. [72] that contained two parts. In the first part, PCA was used to rank the top CpG markers based on their predictive ability for lung cancer. The top CpG markers were then used to generate predictive algorithms for six different AI models, including RF, SVM, LDA, Prediction Analysis for PAM, GLM, and ANNs like as previous study. In the second part, discriminative CpG markers are identified in two parts of the genome, coding genes and nonprotein coding DNA regions. The DL model achieved exceptional results, boasting a perfect AUC of 100%, along with an impressive sensitivity of 95% and a specificity of 100%.

A diagnostic prediction model based on DNA and cfDNA methylation data was applied to 27 cancer types of TCGA database. To achieve this, they used moderated t-statistics to identify the most differential methylation expression between whole-genome and promoters’ CpGs. They then utilized two ML methods, Lasso and RF, to recognize the markers with the biggest methylation beta value difference. Finally, two multilayer FNNs were implemented to distinguish between cancer samples of different subtypes and normal samples. The performance of the method was evaluated based on the detection of cancer from methylation data in tissue and cfDNA. The findings demonstrate that the method consistently delivered outstanding performance in cancer detection, boasting an impressive average across key metrics AUC, accuracy, sensitivity and specificity of 98, 91, 93.65 and 92.8%, respectively, for both tissue and cfDNA samples [54].

### Estimating patient survival and survival-sensitive subtypes in cancer

#### Multi-omics datasets

A novel computational method, known as ML_ordCOX [55], was designed to explore the relationship between GE and DNA methylation in breast cancer and its impact on patient survival. This method comprised several stages, including gene co-expression clustering, the main and auxiliary BLSTM network stages, and the Cox model stage. The main BLSTM network consisted of multiple layers, such as the BLSTM layer, time-distributed layers, dropout layers and FC layers, which were utilized to predict patient survival risk. Additionally, the auxiliary network had one BLSTM layer and one FC layer to obtain the ordinal loss, which calculated the ordinal survival information among different patients. The performance of the ML_ordCOX method was assessed, and the evaluation yielded a C-index value of 0.72, indicating its potential as a powerful and effective tool for breast cancer prognosis.

The stacked denoising AEs (SdA) [56] is a computational method used to classify subtypes of kidney renal clear cell carcinoma (KIRC) with discriminative survival probability using multiplatform genomic datasets. This method integrated five genomics datasets in order to distinguish two subtypes from KIRC patients. The SdA model was used to learn a high-dimensional representation of the genomic data and capture the underlying patterns and relationships between features. It separately allocated one hidden layer and two hidden layers for (miRNA, protein) and (DNA methylation, GE, CAN), respectively. Then, it combined the last individual hidden layer from each dataset as the input layer for FC layers as last joint hidden layer which were built from three hidden layers. According to the hidden values from FC layers, the KIRC patients were identified into two groups using *K*-means clustering method. The results showed that the SdA method was effective in distinguishing KIRC subtypes with *P*-value = 4.37 × 10^−7^, and average Pearson correlation was 0.13 between the original input and the reconstructed input from LJHL.

A DL-based model was developed to predict the prognosis of lung adenocarcinoma patients using multi-omics data. The method consisted of two parts. In the first part (training), the model stacked the four unit-norm scaled matrices and used them as input for an AE to extract new features. Univariate Cox proportional hazard (Cox-PH) models were then applied to these new features to identify those significantly associated with overall survival (OS). A covariate Cox-PH model with Lasso regression was performed to select features by minimizing the log partial likelihood. Survival subgroups were identified using *K*-means clustering based on the reduced features associated with survival. In the second part (robustness validation), DEGs between survival subgroups were calculated using DEseq2 and ANOVA *F*-values methods for each omics data type, and random forest models were constructed using these genes. The performance of the model was evaluated on validation dataset with log-rank test *P*-value 9.3 × 10^−3^ and C-index value of 0.64 [57].

The Multi-view Factorization Autoencoder (MFAE) method [58] was designed to improve the understanding of bladder urothelial carcinoma and brain lower grade glioma by integrating multi-omics data and biological interaction networks. The method incorporated various data types, such as DNA methylation, miRNA expression, protein expression and clinical data, to capture a comprehensive view of the diseases. Using multiple factorization AEs as submodules for each omics data type, the MFAE model employed DL techniques to identify relevant features and integrate the multi-omics data with biological networks. By fusing different views in a latent space, the model enhanced the representation power and incorporated prior knowledge of biological interactions. Additionally, the model simultaneously trained molecular data and patient embeddings to predict clinical outcomes, including Progression-Free Interval and OS events, offering valuable insights into disease progression and patient survival. The MFAE method demonstrated promising results for both bladder urothelial carcinoma and brain lower grade glioma, showcasing average AUC values of 78% and average precision values of 70%.

The i-Modern methodology [59] developed an integrated matrix to represent high-dimensional multi-omics features for each patient to discovery significant multi-omics signatures that contribute to the stratification of glioma patients. This matrix was created using AE DL technology to transform complex multi-omics data into low-dimensional features. i-Modern incorporated clinical information and employed a univariate Cox-PH model to select informative features based on their significance (Wald and Log-rank test *P*-value < 0.05). *K*-means clustering was used to group samples based on these informative features. The Wilcoxon test was applied to continuous features and the Chi-square test or Fisher’s exact test to categorical features to determine the multi-omics features most correlated with a patient's risk subgroup. The top-ranked features were used to construct multiple prediction models, including FC layers, SVM, RF, LogitBoost (LGB) and Naive Bayes (NB) with optimized parameters determined through a grid search. This method achieved an impressive AUC of 99% for multi-omics data, and it demonstrated high accuracy 98%, sensitivity 99% and specificity 96% for the FC layers prediction model.

AE is a model to integrate DNA methylation, RNA-seq and miRNA-seq data in CRC patients. The AE model concatenated the three data matrices and reduced their dimensionality into a lower dimensional representation. The transformed features were then evaluated using a COX-PH model to assess their predictive effect on the hazard rate. Using *K*-means clustering, the tumor samples were divided into two groups based on survival-related features. The resulting clusters (G1 and G2) showed distinct survival rates, suggesting their potential for predicting survival outcomes in CRC patients. The evaluation yielded a C-index value of 0.78 and log-rank test *P*-value of 1.53 × 10^−7^, indicating its potential as a powerful and effective tool for CRC cancer prognosis [60].

Utilizing AEs, the correlation between the LUAD and LUSC subtypes of non-small cell lung cancer (NSCLC) and survival outcomes has been explored [61]. The data were preprocessed and input samples were divided into two datasets based on common and uncommon sample IDs. AEs were then applied to reduce the number of features in each omics data matrix. The selected features were standardized and analyzed using the COX-PH model to identify statistically significant features associated with patient survival. These features were merged into a final data matrix. ML techniques, including k-means clustering and logistic regression, were employed to identify subtypes of lung cancer based on predicted survival outcomes. Additionally, XGBoost and LightGBM models were used to determine the proteins closely associated with these subtypes. The importance of each feature in determining the subtypes was assessed using the SHAPley value. The results of the logistic regression models for predicting survival subtypes showed large variation with AUC scores ranging from 43 to 99% for each omics data matrix. *K*-means clustering with *K* = 10 showed a significant difference in survival rates (log-rank test: *P* = 0.003) too [65].

An AE-based model [62] was developed to identify survival-sensitive subtypes in Gliomas tumors. RNA-seq and DNA-methylation data from the TCGA were preprocessed to generate input features for the AE model. From AE's bottleneck layer, 100 new features were selected, and 46 of them were found to be significantly associated with survival based on a univariate Cox-PH model. K-means clustering was applied to these 46 features, revealing that the optimal number of clusters was 2. These resulting subtypes were used as labels for training an SVM model with cross-validation. The SVM model incorporated the top 100 mRNAs and 100 methylation features, selected through ANOVA feature ranking. By utilizing the AE model and SVM classification, this study aimed to identify distinct subtypes in Gliomas tumors that are particularly sensitive to survival, offering insights into personalized treatment approaches. The performance of the method to estimate the two subtypes using the integrated RNA-seq and DNA methylation data was found to have a C-index of 0.92, a log-rank *P*-value of less than 0.0001 and a Brier score of 0.16.

Two principles, known as ConcatAE and CrossAE, were introduced to integrate multi-omics data based on using multiview learning approach [63]. ConcatAE involves the training of independent AEs for each modality, followed by concatenation of hidden features from each modality, and finally feeding them into a task-specific network. CrossAE involves training an AE for each modality independently, followed by cross-modality translation to reconstruct input features from other modalities, allowing consensus representation among modalities. The study’s final step involved combining hidden features from each modality with element-wise average and training encoders and task-specific networks with task-specific loss functions such as the cross-entropy loss for classification or the negative partial log-likelihood loss for survival regression. These two principles provide effective methods for integrating multi-omics data and have the potential to improve the performance of multiview ML models. The performance of integration DNA methylation and miRNA expression for predicting the survival outcome of breast cancer patients using ConcateAE and CrossAE approaches was found to have a C-index of 0.64 and 0.63, respectively.

#### Unimodal data (DNA methylation)

The process of developing a deep survival Epigenome-Wide Association Study (EWAS) method [64] was designed to discover DNA methylation patterns in blood associated with the duration between the onset of a disease and its detection or diagnosis (Time to Diagnosis) that would enhance the relationship between DNA methylation patterns and survival outcomes in breast cancer. The method consisted of multiple stages, starting with a feature agglomeration analysis on CpG island methylation profiles to identify profiles significantly associated with Time to Death (TTD) outcomes. A novel DL-based approach was then developed to model the complex relationship between the aggregated features and TTD in breast cancer survival analysis. In the final stage, the importance of each feature was estimated using Shapley Feature Attribution methods, specifically the Shapley value, to determine their relative significance in predicting TTD. The performance of the deep survival EWAS model was evaluated on breast cancer patients yielding a high C-index = 0.70.

### Overview of DL model similarity

An alternative categorization approach has been implemented for a total of 35 research articles, which explored diverse DL architectures, based on the similarity of their DL models. These articles were subsequently grouped into nine distinct categories. A concise summary of the outcomes from this categorization is presented in Table 5, encompassing details such as the DL model, categorization, complexity, multi-omics integration, benefit derived from the utilization of DL models, and performance.

**Table 5:** An overview of the categorization of selected studies based on model similarity

Ref.	DL Model^a^	Categorization^b^	Complexity^c^	Multi-omics integration^d^	Benefit derived from the utilization of DL Models	Performance
Feedforward Neural Network/ Fully Connected Hidden Layers/ DNN/ MLP/ ANN						
[39]	FNNs	i	**	Early integration	Conducting LR analysis to predict GE patterns based on DNA methylation data, and employing FNNs for classification to assess the labels of genes exhibiting differential expression.	AUC = 85%
[43]	DNN	i	**	Early integration	The GCBMI model integrates a DNN as a classifier to evaluate DEGs in conjunction with DNA methylation data.	AUC = 98%
[45]	Sequential DNN	i	**	—	Create a sequential DNN as classification approach for cancer prediction.	AUC = 98.75%
[46]	DNN	i	**	—	Conduct a DNN coupled with a stacking ML algorithm for DNA methylation-based classification.	AUC = 100%
[53]	ANNs	iii	**	—	To classify samples, ANNs was developed using statistically significant differentially methylated CpGs as discriminative features.	AUC = 100%
[53]	ANNs	iii	**	—	ANNs is developed to classify samples based on discriminative CpG markers found in both coding and noncoding regions	AUC = 100%
[44]	MLP	i	***	—	Binary and multiclass DNN classification models were trained to distinguish malignancy in samples and perform pan-classification for specific cancer diseases, utilizing shared features.	AUC = 90.92%
[50]	FC layer	i	***	—	Two FC layers are employed for classification purposes, with one of them distinguishing between healthy and cancerous groups, while another DL model was utilized to estimate cancer types.	AUC = 99.68%
[52]	MLP	i	***	—	Create a DNA methylation-based network MLP models for classification holed promise in addressing both the diagnosis of cancer of unknown primary origin and the identification of cancer cell types in circulating tumor cells.	Sensitivity = 94.48% Specificity =99.80%
[73]	FC layer	ii	***	—	Create an FC layer as a regression task aimed at predicting the target genes' promoter methylation profiles using landmark genes as input.	MAE = 0.03 PCC = 0.82
[54]	multi-layer FNNs	iii	***	—	To build two multi-layer FNNs as diagnostic prediction models for sample classification, utilizing CpG methylation and promoter markers as input features.	AUC = 98%
[64]	multi-layer FNNs	iv	***	—	Create a deep survival model using multilayer FNNs that leverage aggregated features to capture the intricate relationship between features derived from CpG islands and specific cancer types.	C-index =0.70
AE and its variants						
[41]	DAE+ MLP	i	**	Early integration	Using DAE for dimensional reduction and employing MLP for both supervised classification for predicting class labels and as a multi-output regressor for estimating RNASeq,	AUC = 95.1% Correlation coefficient = 0.68
[48]	VAE	i	**	—	Creating a multitask model for diverse DNA methylation data with varying targets across a range of tasks.	Accuracy =97%
[51]	VAE	i	**	—	Distinguishing between original subtypes within a particular cancer type can be achieved by applying a VAE model to DNA methylation data. This approach allows for the extraction of a biologically significant latent space that captures essential characteristics of cancer samples.	AUC = 91.50%
[38]	CVAE+ FC layer	i	***	Early integration	CVAE offers unique benefits when delving into the intricate landscape of genomic and epigenomic data, surpassing other feature extraction techniques.	AUC = 91.19%
[66]	VAE	ii	***	Early integration	Create a multitask model to impute two aspects of missing data: data missing completely at random (MCAR) and data missing not at random (MNAR)	Root mean square: (GE, DNA Methylation) = (0.31,0.70)
[56]	Stacked denoising AEs	iv	***	Early integration Intermediate integration	SdA merged input datasets with varying numbers of features, resulting in a significantly improved separation of patients based on clinical outcomes.	C-index =0.72
[57]	AEs	iv	***	Early integration	The model incorporates a sequence of AE, univariate Cox-PH analysis and LASSO regression as feature extraction to identify discriminative features for categorizing survival subgroups.	C-index =0.64
[59]	AE, FC networks	iv	***	Early integration	i-Modern has regarded comprehensive multi-omics datasets with high-dimensional features. It has undergone optimization and training of an AE to effectively reconstruct multi-omics data through complex functions and define selected the top ranked multi-omics features as landmark signatures.	AUC = 99%
[60]	AE	iv	***	Early integration	Identify survival-related multi-omics features based on a sequence of AE, univariate Cox-PH analysis.	C-index =0.78
[61]	AE	iv	***	Intermediate integration	Perform a sequence of AE-based, univariate Cox-PH on multi-omics datasets to identify multi-omics attributes relevant to survival.	AUC = average [43%–99%] =60.83%
[62]	AE	iv	***	Early integration	Detect survival-relevant multi-omics attributes through a series of AE-based, univariate Cox-PH analyses.	C-index =0.92
[42]	Stacked AEs + DFNForest	i	****	Intermediate integration	DFNForest optimizes dedicated layers for data modalities, improving predictive feature extraction. This model is an innovative ensemble of FNT models for cancer subtype classification.	Accuracy =74.32%
[58]	Multiple AEs	iv	****	Intermediate integration	The MAE model is constructed based on three key principles: matrix factorization, multi-view learning, and DL. This approach is employed to integrate various perspectives within the latent space of each type of -omics data.	AUC = 78%
[63]	AEs	iv	****	Intermediate integration	CrossAE introduced an innovative approach to multi-omics integration, wherein an AE is individually trained for each modality, enabling the reconstruction of input features from different modalities.	C-index = [0.63–0.64]
Capsule Neural Network						
[40]	CapsNet	i	****	Early integration	In the CapsNet architecture, consisting of two convolutional layers and one FC layer, samples can be effectively classified using multi-omics data.	AUC = 94.6%
[47]	CapsNet, MLP, GLRM in DNN	i	****	—	MethylCapsNet is created by building upon CapsNet, and it effectively organizes CpG groups, integrating biologically relevant information into a set of capsules for the classification of DNA methylation patterns.	Accuracy = 98%
Bi-directional LSTM Unite and Convolutional Neural Network						
[49]	CNN+ BiLSTM	i	****	—	The DISMIR model integrates CNNs for local pattern detection and connection analysis among neighboring nucleotides within a fixed window, alongside BiLSTM to manage long-range dependencies in DNA sequences and incorporate DNA methylation data from plasma samples.	AUC = 99.69%
Bi-directional LSTM Unite and Fully Connected Hidden Layer						
[55]	BiLSTMs+ FC layers	iv	****	Intermediate integration	The ML_ordCOX employs a BiLSTM and an ordinal Cox model network to analyze multi-modal data encompassing gene mRNA expression and DNA methylation. It leverages this information to predict survival outcomes for a particular cancer type.	C-index =0.72
[68]	BiLSTMs+ FC layers	ii	****	—	The BiLSTM-5mC model incorporates two separate BiLSTMs, each followed by FC layers, to implement two distinct feature encoding method, namely One-hot and NPF, and FC layers as regression tasks to predict genome-wide DNA methylation.	AUC = 96.35%
Convolutional Neural Network						
[70]	CNNs	ii	****	—	The MRCNN model is constructed based on a sequential CNNs with different size of convolution kernel as methylation regression approach for prediction of genome-wide DNA methylation.	AUC = 93.2%
Bi-directional Gated Recurrent Unit and Convolutional Neural Network and Fully Connected Hidden Layer						
[67]	CNNs +BiGRUs + FC layers	ii	*****	—	The DeepCpG model comprises three key modules: CNNs, BGRUs, and FC layers. CNNs are responsible for extracting local and meaningful features from the DNA sequence. BGRUs, on the other hand, excel at recognizing long-term dependencies within the CpG neighborhood across all cells. To tie it all together, FC layers play a pivotal role by concatenating the outputs from both the CNNs and BGRUs.	AUC = 92.4%
Convolutional Neural Network and Transformer						
[69]	CNNs+ Transformer	ii	*****		DNA sequence around CpG sites as input data for CpG transformer encoder is processed using CNNs. Multihead attention mechanisms in transformer encoder has been adapted to utilize sliding window row and column attention, enhancing the model's ability to capture interactions between neighboring CpG sites by incorporating axial attention for prediction of genome-wide DNA methylation.	AUC = 91.68%
Convolutional Neural Network and Transformer and Fully Connected Hidden Layer						
[71]	CNNs+ Transformer+ FC layers	ii	*****	—	The INTERACT model is built upon three primary modules: CNNs for detecting local DNA sequencing and DNA methylation patterns encoded through one-hot encoding, a transformer encoder to capture important features and long dependencies, and finally, using FC layers as regression tasks to predict genome-wide DNA methylation.	AUC = 82.5%
^a^DL models: Artificial neural network (ANN) Convolutional neural network (CNN) Deep autoencoder (AE) Recurrent neural network (RNN) Long-short-term-memory (LSTM) Gate recurrent unit (GRU) Capsule neural network (CapsNet) Transformer Convolutional Variational Autoencoder (CVAE) Feedforward Neural Network (FNN) Fully Connected Hidden Layer (FC layer) Denoising Autoencoder (DAE) Multi-layer Perceptron (MLP) Deep flexible neural forest (DFNForest) Deep Neural Network (DNN) Deep-Learning analog of a Group LASSO Regression Model (GLRM in DNN) Variational Autoencoder (VAE) Bi-directional long Short-Term Memory Unite (BiLSTM) Bi-directional Gated Recurrent Unit (BiGRU)						
^b^Four categorization groups: Cancer epigenetics subtype classification Missing value imputation and prediction of CpG methylation data Identification of epigenetic biomarkers for cancer diagnosis and prognosis Estimating patient survival and survival-sensitive subtypes in cancer						
Multi-omics datasets: RNA sequencing mRNA sequencing microRNA sequencing Gene expression DNA copy number variation Protein expression DNA methylation DNA sequencing (somatic mutations) Unimodal data: DNA methylation data Clinical patient information						
^c^The complicity of DL model encompasses various aspects, including architecture, parameters, data preprocessing, hyperparameters, regularization, computational demands, optimization, ensemble methods, interpretability, and the use of pretrained models. ^d^Early integration and intermediate integration are two ways to integrate multiple data modalities.						

It is worth noting that terms such as FNN, FC layer, DNN, MLP and ANN, while often interconnected, signify distinct aspects and variations within the realm of neural network models. These models find primary application in DNA methylation analysis, categorized into two main domains: multi-omics datasets and unimodal data. Within these domains, they serve purposes ranging from binary and multiclass classification tasks aimed at label recognition to regression tasks focused on predicting target values. The performance metrics reveals varying degrees of success among the studies. In the classification domain, AUC values range from 85% to a remarkable 100%, suggesting diverse levels of discriminative power in the models developed. In contrast, for regression tasks, the MAE is 0.03 and a C-index of 0.70.

The AE and its variants such as DAE, VAE, CVAE, multiple AEs and stacked denoising AEs or AEs are considered as a group of deep AEs initially designed for dimensionality reduction. They were primarily developed for applications in two key domains: multi-omics datasets and unimodal data. Deep AEs offer the flexibility to incorporate multiple data modalities, allowing the exploitation of complementary information. This integration can be achieved through two main approaches. The first method involves early integration, where features from each dataset are concatenated at an initial stage. The second approach is intermediate integration, wherein each data modality undergoes dedicated processing layers. Subsequently, the outputs of these layers are concatenated, followed by further layers for the integration of features extracted from each data modality. The performance metrics exhibited a range of values, including AUC (60–99%), C-index (0.63–0.92) and accuracy (74–97%).

The CapsNet model is utilized either in isolation or in conjunction with other DL models for classification tasks. The performance of this specific DL model type, applied to both multi-omics datasets and unimodal data, yielded reported metrics of 94.6% for AUC and 98% for accuracy, respectively.

Ensemble models based on DL architectures, such as BiLSTM, BiGRUs and transformerare all DL architectures commonly used for sequential data processing, are employed in tandem with other DL models such as CNNs for pattern detection in sequencing data or FC layers for regression tasks, and even for integrating the output of other modules. These categories exhibit a notably higher level of complexity compared with other groups. While BiLSTM, BiGRU and Transformer are all used for sequential data processing, they differ in terms of architecture, parallelization, attention mechanisms and parameter efficiency. These models play a pivotal role in identifying long-term dependencies within CpG neighborhoods or DNA sequencing. In addition, the performance metrics exhibited a range, with AUC values spanning from 82 to 99%, with a C-index of 0.72.

#### Limitations of DL

The success of DL-based methodology in processing of DNA methylation data is of particular importance because these approaches has the potential to be applied in a wide range of biological processes, including chromosome instability, X-chromosome inactivation, cell differentiation, cancer progression and gene regulation [74]. Consequently, researchers are actively designing and using DL networks for corresponding tasks. Several DL architectures have been employed in DNA methylation analysis and multi-omics datasets. These architectures are shown for four categories. While DL architectures have demonstrated their effectiveness in various aspects of DNA methylation analysis, such as classification, feature extraction, imputation, biomarker identification and survival analysis, there are major limitations in processing DNA methylation data and multi-omics data. In the following section, we will discuss these limitations in detail.

Black boxes: DL architectures are often criticized for lacking transparency in how they produce outputs. In the field of bioinformatics, it is crucial to interpret these models to comprehend the biological and computational patterns they detect. Consequently, there is a rising preference for ‘white-box’ approaches that offer more interpretability and transparency. By enabling interpretation, researchers can gain a deeper understanding of the biological relevance of the results, which enhances the usefulness and dependability of DL models in bioinformatics research.

Overfitting: DL architectures have a complex nature with numerous interconnected parameters. To tackle this issue, several techniques have been developed, including dropout, batch normalization, weight decay, data augmentation and data corruption. These methods aim to improve model performance and generalizability, especially when training data are limited. Insufficient training data are identified as the primary cause of overfitting, and the mentioned techniques address this challenge by modifying model parameters, architecture and manipulating input data.

High dimensionality and low sample size: This issue poses significant challenges for DL. The data requirements of DL models are significantly higher than those of other ML algorithms, making access to more data crucial for achieving optimal performance. Collaborative data repositories can be an effective solution for addressing this challenge. However, it is important to consider the appropriate algorithm type and performance measures during data collection can help to avoid erroneous data interpretations.

Imbalanced data: Imbalanced datasets are characterized by an uneven distribution of observations among the target classes, meaning that one class label has a notably higher number of observations, while the other exhibits a significantly lower count. Training DL models using imbalanced data can lead to undesirable results. Fortunately, transfer learning can offer a viable solution for addressing the class imbalance problem. By initially training the model using a general dataset, transfer learning can effectively mitigate the challenges associated with imbalanced data in epigenetic data analysis.

Fine-tuning a DL model: The issue is a complex process that requires careful analysis of initial results, as it depends on the dataset and research question. Hyperparameters, including learning rate, batch size, momentum and weight decay, are critical for every DL architecture and can be adjusted during training. These hyperparameters control various aspects of the model, such as step size, number of training samples per iteration, optimization of training paths and weight multiplication after updates. It is crucial to correctly set these hyperparameters to avoid under-fitting or over-fitting problems.

Limitation of resource-constrained environments: This issue poses significant challenges due to the computational complexity and memory requirements of DL models. To address this, novel hardware solutions such as Graphics Processing Units and Field Programmable Gate Arrays have been proposed, alongside model compression techniques. These compression methods encompass diverse approaches including parameter pruning, knowledge distillation, compact convolutional filters and low rank factorization, all aimed at reducing the computational overheads of the models.

Balancing accuracy and interpretability: the application of ML and DL methods in studying epigenomics can aid in explaining the underlying mechanisms of diseases and developing new treatments. However, many of these methods prioritize accuracy or efficiency over interpretability, hindering our ability to understand the relationship between epigenomic markers and diseases. This limitation can restrict the use of ML and DL in clinical settings where decisions rely on transparent reasoning. Therefore, it is crucial to establish clear reasoning behind any computational model before employing it in high-stakes clinical situations [75].

#### Advantages and future prospects of DL

DL methods are nonlinear algorithms that are highly flexible and can identify patterns in complex, high-dimensional data. These methods can model data more accurately than most other classical ML algorithms and are used for dimensionality reduction, clustering and identifying correlations between data types. A key benefit is the ability of DL methods to integrate multiple steps of data processing into a single model, saving time and minimizing errors. Another advantage is its capacity to incorporate various types of data, such as electronic health records, molecular data, digital pathology and radiographic images, enabling a comprehensive understanding of the heterogeneity seen in complex diseases. This integration facilitates tailoring medical care and advancing personalized medicine [76]. Finally, DL makes it easier for researchers with limited experience with mathematics or statistics to use sophisticated approaches by applying streamlined tools.

DL will continue to be a crucial tool in the diagnosis, screening, treatment and prognosis of disease, including cancer. Emerging application areas, for example, include drug discovery and pharmacogenomics [77]. The future of cancer epigenetics research, however, will require the on-going development and deployment of cutting-edge DL architectures. Recent advances in DL, specifically in large language models built on transformer and self-attention architectures, along with the availability of extensive and cost-effective data, hold great promise. For example, transformer neural network architecture can be used to analyze combined DNA sequence and methylation data obtained from plasma cfDNA. Such tools will also bring us closer to achieving the goal of precise biomolecular experimentation in a virtual setting.

## CONCLUSION

In this work, we first introduced current state-of-the-art DL architectures. We then reviewed the current state of the DL methods and its application in cancer epigenetics, specifically those involving DNA methylation data. This review provides a comprehensive overview of recent advances in DL-based approaches in four categories: diagnosis or classification of cancer epigenetics and diseases, prediction of CpG methylation states and imputation of missing values, biomarker discovery in tissue and cfDNA samples, and estimation of patient survival and survival-sensitive subtypes in cancer. We also discussed the challenges in processing DNA methylation data and multi-omics data using DL methods, including interpretability, overfitting, the curse of dimensionality, imbalanced data, parameter and hyperparameter tuning, the deployment of DL models in resource-constrained environments, and the attention to improving accuracy which sometimes lacks transparency in the clinical context. Finally, we highlighted emerging DL tools that are likely to play a key role in future DNA methylation analyses.

**Key Points**

- Deep learning (DL) methods are increasingly being used to facilitate analyses of genome-wide DNA methylation patterns.
- DL methods have been applied to multi-omic datasets and unimodal data to inform biomarker discovery, classification of disease status, data imputation and survival analysis.
- DL methods have shown impressive performance on these tasks, but successful application depends critically on the DL architecture.
- The challenges of DL include interpretability and overfitting, as well as the technical issues of parameter tuning and dealing with imbalanced datasets.
- The ongoing and rapid development of DL, for example in large language models, heralds a promising future for the analysis of DNA methylation data and its application to clinical genomics and precision oncology.

## Supplementary Material

supplementary_files_bbad411
