# Supplementary material for: Application of deep learning in cancer epigenetics through DNA methylation analysis
Source: Brief Bioinform. 2023 Nov 20;24(6):bbad411. doi: 10.1093/bib/bbad411 (PMC10661960; doi:10.1093/bib/bbad411)
Supplement: supplementary_files_bbad411 [file supplementary_files_bbad411.docx]

**Application of deep learning in cancer epigenetics through DNA methylation analysis**

**Maryam Yassi ^1,2^, Aniruddha Chatterjee ^*2, *3^, Matthew Parry ^*1, *4^**

^1^Department of Mathematics and Statistics, University of Otago, Dunedin, New Zealand.

^2^Department of Pathology, Dunedin School of Medicine, University of Otago, Dunedin, New Zealand.

^3^Honorary Professor, UPES University, Dehradun, India.

^4^Te Pūnaha Matatini Centre of Research Excellence, University of Auckland, Auckland, New Zealand.

*Joint senior authors.

1. **Deep Learning Architectures**

With the significant improvement of computational power and the advancement of big data, DL has become one of the most successful ML algorithms to deal with the explosion of data in many fields like genomics and next generation sequencing in recent years (Schmidt and Hildebrandt 2021). Furthermore, DL algorithms can be regarded both as a sophisticated and mathematically complex evolution of ML algorithms. DL describes algorithms that analyse data with a logical structure similar to how a human would draw conclusions. Note that this can happen both through supervised and unsupervised learning. To achieve this, DL applications use a layered structure of algorithms called an artificial [neural network (ANN)](https://levity.ai/blog/neural-networks-cnn-ann-rnn). The design of such an ANN is inspired by neurons in the human brain, and transmit information through layers of weighted, interconnected computational neurons (Zurada 1992).

- 1. **Artificial Neural Network**

ANN is very powerful classifiers; it has been mathematically proven that an ANN can learn any mathematical function to arbitrary precision, given enough training time and sufficient data. ANN is built up of nodes, representing neurons, and connections between nodes, representing axons and dendrites carrying information in the human brain. Connections in an ANN is weighted. The neurons are organised in three layers in a network architecture, the input layer that representing one particular input data vector, the hidden layer (more than one), and the output layer which providing a result of the classification. The hidden layer plays a main role in extracting some of the most relevant patterns from the inputs and sends them on to the next layer for further analysis. It accelerates and improves the efficiency of the network by recognizing just the most important information from the inputs and discarding the redundant information. The simplest and by far the most widely used form of ANN is the [perceptron](https://www.sciencedirect.com/topics/immunology-and-microbiology/perceptron), a fully connected feed-forward network or multilayer perceptron (MLP) is demonstrated in Figure 3.A. Each node in the hidden and output layers calculates the weighted sum of its inputs to determine the final input value. For a given node (j) the output value of each of the nodes (i) in the last layer connected to node (j) is multiplied by the weight $(w_{ij})$ on that connection. The values of all of the inputs are summed, $y_{j}$=$\sum_{i=1}^{n} x_{i}w_{ij}$. This value is then modified using a nonlinear function known as the activation or transfer function to produce the node’s output value.

- 1. **Convolutional Neural Network**

Convolutional [neural network](https://www.sciencedirect.com/topics/computer-science/neural-networks) (CNN) (Krizhevsky, Sutskever et al. 2017)  is the first [DL models](https://www.sciencedirect.com/topics/computer-science/deep-learning-model) that received a lot of attention due to their impressive performance in applications of computer vision. The main idea behind CNN is to extract local features from the data. They consist of three main types of layers, which are convolutional layer, pooling layer, ffully-connected layer (FC layer) (Figure 3.B). Convolution is a mathematical operation that allows the merging of two sets of information. In the case of CNN, convolution is applied to the input data to filter the information and produce a feature map. In fact, during convolution operation a $n\times m$kernel (n > 0 and m > 0) scans the input data to automatically extract features. Moving across the input data based on a stride value to generate a feature map. Pooling (or down sampling) is carried out on the feature map to ensure that the CNN recognizes the same object in images of different forms and also to reduce the memory requirements of the model. It introduces spatial invariance in CNN; which eventually turns out to be one of the major weaknesses of CNN. There are several types of pooling including max pooling, min pooling, average pooling. FC layer performs the task of classification by fully connected network and based on the features extracted through the previous layers and their different filters. While convolutional and pooling layers tend to use ReLu function, FC layers usually leverage a softmax activation function to classify inputs appropriately, producing a probability from 0 to 1.

- 1. **Deep Autoencoder**

Autoencoder (AE) (Baldi 2012) is unsupervised ANN that is designed to learn efficient data encoding and representation to reconstruct the original input data. As shown in Figure 3.C, an AE consists of two parts: an encoder and a decoder. The encoder is used to generate a reduced feature representation from an initial input $x$ by a hidden layer h. The decoder is used to reconstruct the initial input $z$ from the encoder's output by minimizing the loss function $l(x,z)$ . The AE converts high-dimensional data to low-dimensional data. Therefore, the autoencoder is especially useful in noise removal, feature extraction, compression, and similar tasks. There are four types of autoencoder: Denoising Autoencoder (DAE) (Vincent, Larochelle et al. 2008) , the Sparse Autoencoder (Huang, Zhang et al. 2020), Contractive Autoencoder (CAE) (Rifai, Bengio et al. 2012) and Variational Autoencoder (CAE) (Doersch 2016).

- 1. **Capsule Neural Network**

Capsule neural network (CapsNet) is a relatively new type of neural network architecture that was first developed in 2017 (Sabour, Frosst et al. 2017) to address some of the limitation of CNN. During the process of max pooling in CNN, lots of important information is lost because only the most active neurons are chosen to be moved to the next layer. This operation is the reason that valuable spatial information gets lost between the layers. Furthermore, the major challenge of CNN that led to the introduction of CapsNet is their inability to recognize new viewpoints like pose, texture and deformations of an input image. Each capsule is a group of neurons whose activity vector represents the instantiation parameters of a specific type of entity such as an object or an object part. As shown in Figure 3.D, CapsNet consists of three layers: Conventional layer, primaryCaps and classCaps layers. In procedure of conventional layer, the initial feature matrix of input data is converted to higher-level and more abstract local features. For example, if 256 filters are applied in the convolutional layer, then there are 32 channels of 8D capsules, each capsule is a vector contains 8 convolutional units in primaryCaps layer. The output length of a capsule represents the probability that the entity exists and is defined by a non-linear weighting function, called a squashing function. This function ensures that the length of the output from the capsule lies between 0 and 1. In the classCaps layer are usually composite entities of primaryCaps layer, and this could be responsible for doing classification and defining target class which a prediction is made based on the length of the classCaps. Capsules in lower layer route their output to appropriate capsules in next layer based on “Dynamic routing algorithm” which is an iterative procedure and it dose in multiple steps between layers. This is performed by considering similar orientation and magnitude between capsules for transferring information from lower layer to higher layer.

- 1. **Recurrent Neural Network**

A major characteristic of all neural networks is that they have no memory. Each input shown to them is processed independently, with no state kept in between inputs. Recurrent neural network (RNN) is a powerful and robust type of neural network, and belong to the most promising [algorithms](https://builtin.com/data-science/tour-top-10-algorithms-machine-learning-newbies) in use because it is the only one with an internal memory. Because of their internal memory, RNN can remember and capture important information about the input they received, which allows them to be very precise in predicting what’s coming next. This is why they’re the preferred algorithm for sequential data like [time series](https://builtin.com/data-science/introduction-segmentation-correlation-time-series-modeling), speech, text, DNA sequences (Williams and Zipser 1989) (Liu, Qiu et al. 2016). In RNN, a feedback loop connects the output from step t-1 to the network and influences the outcome of step t (Figure 3.E). This feedback loop differentiates the RNN from the regular feed forward networks.

The process of carrying memory forward is mathematically represented in Eq. (1):

$S_{t}=tanh\left( {{W_{s}S}_{t-1},W_{x}X}_{t} \right) , Y_{t}={W_{y}S}_{t} (1)$

Where $S_{t}$ is the hidden state at time step t, $X_{t}$ the input and $Y_{t}$the output at the same time step, $W_{x}, W_{s},W_{y}$ denote weight parameters for input, hidden state and output, respectively. Moreover, $Tanh$ is activation function. Bidirectional recurrent neural network (BRNN) (Schuster and Paliwal 1997) is another type of RNN that simultaneously learn the forward and backward directions of information flow. This is different from standard RNN, which only learn information in one direction. The process of both directions being learned simultaneously is known as bidirectional information flow. In a typical ANN, the forward projections are used to predict the future, and the backward projections are used to evaluate the past.

- 1. **Long Short-Term Memory Unite**

Long short-term memory network (LSTM) is an advanced RNN (Hochreiter and Schmidhuber 1997) and provides a well-constructed structure by establishing gates in its basic unit which is named as cell. It can solve the problem of vanishing and exploding gradients by proposing a different architectural approach to [RNN](https://www.sciencedirect.com/topics/engineering/recurrent-neural-network). These gates can capture both the long-term memory and short-term memory along the time steps. The LSTM introduces three types of gates which are input gate, output gate, and forget gate. The input gate combines the input and updates the S vector, the output gate combines the current and previous S vectors and the forget gate prevents the current S vector from updates to the given RNN unit. The detail structure of LSTM presented in (Figure 3. F) and the following equations describe the behaviour of the LSTM:

$f_{t}=\sigma(W_{f}S_{t-1}$*+*$W_{f}X_{t})$*,* $I_{t}=\sigma(W_{i}S_{t-1}$*+*$W_{i}X_{t})$*,* $O_{t}=\sigma({W_{o}S}_{t-1}$*+*$W_{o}X_{t})$

$\tilde{c}_{t}=tanh({W_{c}S}_{t-1}$*+*$W_{c}X_{t})$ *,* $C_{t}=(I_{t}\tilde{c}_{t}$*+*$f_{t}C_{t-1})$

$S_{t}=(o_{t}\tanh{(C}_{t}))$ (2)

Here $f_{t}, I_{t}, O_{t}$ are Forget, Input and Output gates, respectively; $X_{t}$ and$o_{t}$ are an input and output vector and $S_{t}$ is the hidden state; $\tilde{c}_{t}$ and $C_{t}$ are cell input and cell state vectors;$W_{f}, W_{i}$*,* $W_{o},W_{c}$ denote weight parameters for forget, input, output and cell content, and $\sigma,tanh$ are activation functions. In Bidirectional Short-Term Memory Unites (BLSTMs), the input flows in both directions, and it’s capable of utilizing information from both sides. It’s also a powerful tool for modelling the sequential dependencies in both directions of the sequence. BLSTMs adds one more LSTM layer, which reverses the direction of information flow. Briefly, it means that the input sequence flows backward in the additional LSTM layer. Then the outputs are combined from both LSTM layers.

- 1. **Gate Recurrent Unites**

The Gated Recurrent Unit (GRU) (Chung, Gulcehre et al. 2014) is a new type of RNN that, in certain cases, has advantages over long LSTM . GRU uses less memory and is faster than LSTM, however, LSTM is more accurate when using datasets with longer sequences. Similarly, to the LSTM unit, the GRU has gating units that modulate the flow of information inside the unit, however, without having a separate memory cell. GRU also only has two gates, a reset gate and update gate. The update gate acts similar to the forget and input gate of an LSTM. It decides what information to throw away and what new information to add. The reset gate is another gate is used to decide how much past information to forget. The detail structure shown in Figure 3. G and the following equations define the behaviour of the GRU:

$z_{t}=\sigma(W_{z}{.[s}_{t-1}$,$X_{t}]$) , $r_{t}=\sigma(W_{r}{.[s}_{t-1}\text{,}X_{t}]$)

$\tilde{s}_{t}=tanh(W_{s}.[{r_{t}\times s}_{t-1}\text{,}X_{t}]$), $s_{t}=\left( 1-z_{t} \right)\times$ $s_{t-1}+z_{t}$ $\times\tilde{s}_{t}$ (3)

Where $z_{t}, r_{t}$ are update and reset gates.$X_{t}$ and$S_{t}$ are an input and hidden state vectors.$\sigma,tanh$ are activation function. $W_{z}, W_{r}$ denote weight parameters for update and reset gates, and $W_{s}$ is weight parameter for hidden state. The operator $\times$ denotes the [Hadamard product](https://en.wikipedia.org/wiki/Hadamard_product_(matrices)). The Bidirectional gated recurrent Unit (BGRU) (Liu, Wang et al. 2021) model is determined based on the state of two GRUs, which are unidirectional in opposite directions. One GRU that moves forward, beginning from the start of the data sequence, the other GRU that moves backward, beginning from the end of the data sequence. This allows the information from both future and past to impact the current states. The BGRU is defined as follows:

$$\vec{s_{t}}={GRU}_{fwd}\left( X_{t},\vec{s_{t-1}} \right) , s_{t}={GRU}_{bwd}\left( X_{t},s_{t-1} \right)$$

$$s_{t}=\vec{s_{t}} \bigoplus s_{t} (4)$$

Where $\vec{s_{t}}$ is the state of the forward GRU, $s_{t}$is the state of the backward GRU,$\bigoplus$ indicates the operation of concatenating two vectors.

- 1. **Transformer**

Transformer-based models have achieved state-of-the-art performance in various important tasks such as machine translation and question answering. A Transformer model consists of an encoder and a decoder and has an entirely attention-based architecture (Vaswani, Shazeer et al. 2017). As shown in (Figure. 3.H), the encoder part which consists of two sub-layers: the multi-head self-attention layer and the fully connected feed-forward layer. A skip/residual connection structure and layer-wise normalization are incorporated around each sub-layer to greatly facilitate training. The key innovation within encoder is the multi-head self-attention layer which allows the model to associate all the relevant words in a context to encode a specific word better and develop the “contextual understanding” in different aspects. The attention function computes an output based on a query (q) and a set of key-value pairs (K, V). The dot-product attention calculates attention scores by multiplying query with each key and using the products as weights to sum all the values, which can be calculated as:

$$Attention\left( q,K,V \right)= softmax\left( qK^{T} \right)\cdot V=\sum_{i} \frac{exp(qK_{i})}{\sum_{j} exp(qK_{j})}\cdot V_{i} (5)$$

Where $K_{i}$ and $V_{i}$ stands for the i-th key-value pair. Intuitively, the dot-product between $q$ and $K_{i}$ measures the relevance of $V_{i}$ in representing q (how much the model should attend to). Moreover, if we pack a set of queries into a matrix Q, and divide the dot-product by a scaling parameter $\sqrt{d_{k}}$, the scaled dot-product attention is calculated as follows:

$$Attention\left( Q,K,V \right)=softmax\left( \frac{QK^{T}}{\sqrt{d_{k}}} \right)\cdot V (6)$$

Here, $d_{k}$ is the dimension of the keys. The self-attention is a special case of scaled dot-product attention, where Q, K and V come from the same place. Practically, instead of setting Q=K=V and calculate attention scores as $Attention\left( Q,K,V \right)$, it is beneficial to linearly project Q, K and V with different and learnable parameters $W^{Q}, W^{K}$ and $W^{V}$. Then, attention scores can be calculated as $Attention\left( QW^{Q},KW^{K},VW^{V} \right)$. By performing this independently for multiple times, concatenating all the attention scores and once again projected, we get multi-head attention as:

$$MultiHead\left( Q,K,V \right)=Concat\left( {head}_{1}, \ldots,{head}_{h} \right) W^{O}$$

where ${head}_{i}=$ $Attention\left( QW_{i}^{Q},KW_{i}^{K},VW_{i}^{V} \right) (7)$

where $W_{i}^{Q}$, $W_{i}^{K},$ $W_{i}^{V}$ and $W^{O}$ are learnable parameters. Thus, the input and output of each Transformer layer are both matrix of the same shape. Each line of the matrix stands for the representation of its corresponding token.

The raw input of the Transformer is a sequence of tokens. To feed linguistic tokens into a model, the first thing is to transform each of them into a numerical representation. This representation is always called Token Embedding. To achieve this, a vocabulary and an Embedding layer are essential. The vocabulary is a set that contains all the possible linguistic tokens, and the embedding layer $E$ is essentially a learned $n\mathrm{by}m$ matrix where $n$ equals to the vocabulary size and $m$ equals to the customized embedding size. Each line of the matrix stands for the token embedding of a unique token in the vocabulary. The same tokens will be assigned the token embedding. The Embedding layer directly maps a linguistic token in the vocabulary to an $m$-dimensional vector. Since the model has no recurrent or convolutional layers, there is no clear relative or absolute information about the position of the word in the source sentence. In order to let the model, learn the position information better, position encoding is added and superimposed on the word embedding. An encoding method using trigonometric functions maintains its position invariance. The position encoding function can be presented as:

$PE (pos, 2i) =sin(pos/{10000}^{2i/d_{model}}$) (8)

$PE (pos, 2i+1) =cosine(pos/{10000}^{2i/d_{model}}$) (9)

where *pos* is the position of each token; 2*i* and 2*i*+1 are the even-numbered and odd-numbered dimensions of each token position vector of the cardinality, respectively, where all position subscripts start from 0; and $d_{model}$ is the dimensionality of word vector, the same as the dimensionality of encoding.

In the Transformer architecture, the decoder is responsible for generating the output sequence based on the encoder's input representation and the previous output of the decoder. The decoder architecture typically consists of multiple layers of masked self-attention followed by encoder-decoder attention and feed-forward layers. The decoder architecture is very similar to the encoder, but there are a few key differences including Masked Self-Attention. During training, the decoder can only use information from previous positions to generate the current output. This is achieved through a "masked self-attention" mechanism, where the attention mechanism is masked so that each position can only attend to previous positions. The output of the final decoder layer is passed through a softmax activation function to generate a probability distribution over the vocabulary, which is used to select the next word in the output sequence.

| **Table 1: Abbreviations** | |
| --- | --- |
| **Cancer and Disease Epigenetics and DNA Methylation Context** | **Full words** |
| DNA methyltransferases | DNMTs |
| Methyl group | CH3 |
| Whole genome bisulfite sequencing | WGBS |
| Reduced representation bisulfite sequencing | RRBS |
| Methylated DNA immunoprecipitation coupled with next-generation sequencing | MeDIP-seq |
| Differentially methylated regions | DMRs |
| Differentially methylated blocks | DMBs |
| Differentially methylated CPG sites | DMCs |
| RNA sequencing | RNA-Seq |
| mRNA sequencing | mRNA-Seq |
| MicroRNA sequencing , miRNA sequencing | microRNA-Seq, miRNA-seq |
| The cancer genome atlas | TCGA |
| Single-cell bisulfite sequencing | scBS-seq |
| Single-cell reduced representation bisulfite sequencing | scRRBS-seq |
| Circulating cell free tumour DNA | cfTDNA |
| DNA methylation | DNAm |
| Lung adenocarcinoma | LUAD |
| Lung squamous cell carcinoma | LUSC |
| Nucleotide property and frequency | NPF |
| 5-methylcytosine | 5mC |
| Copy number abbreviation | CNV |
| Kidney Renal Clear Cell Carcinoma | KIRC |
| Epigenome wide association studies | EWAS |
| Differentially expressed genes | DEGs |
| Time to Diagnosis | TTD |
| Malformations of cortical development | MCD |
| Central Nervous System | CNS |
| **Artificial Intelligence and Machine Learning Methods** |  |
| Artificial Intelligence | AI |
| Machine Learning | ML |
| Least Absolute Shrinkage and Selection Operator Regression | LASSO regression |
| Support Vector Machine | SVM |
| Random Forest | RF |
| Hierarchical Cluster Analysis | HCA |
| Principal Component Analysis | PCA |
| Principal Component | PC |
| logistic regression | LR |
| Partitioning around medoids | PAM |
| Linear discriminant analysis | LDA |
| Generalized Linear models | GLM |
| local maximum quasi-clique merging | lmQCM |
| Recursive feature elimination | RFE |
| Regression Tree | RT |
| K-Nearest Neighbors | KNN |
| Singular Value Decomposition | SVD |
| Naive Bayes | NB |
| LogitBoost | LGB |
| Prediction Analysis for Microarrays | PAM |
| **Deep leaning Architectures** |  |
| Deep Learning | DL |
| Deep Neural Network | DNN |
| Artificial Neural Network | ANN |
| Convolutional Neural Network | CNN |
| Autoencoders | AE |
| Recurrent Neural Network | RNN |
| Bi-directional Recurrent Neural Network | BRNN |
| Long Short-Term Memory Network | LSTM |
| Bi-directional long Short-Term Memory Unite | BLSTM |
| Gated Recurrent Units | GRU |
| Bi-directional Gated Recurrent Unit | BGRU |
| Capsule Network | CapsNet |
| Convolutional Variational Autoencoder | CVEA |
| Fully Connected Hidden Layer | FC Layer |
| Multilayer Perceptron | MLP |
| Sequential Deep Neural Network | Sequential DNN |
| Deep-Learning analog of a Group LASSO Regression Model | GLRM in DNN |
| Variational Autoencoder | VAE |
| Feedforward Neural Network | FNN |
| Denoising Autoencoder | DAE |
| **Statistical Methods** |  |
| Analysis of variance | ANOVA |
| Uniform manifold approximation and projection | UMAP |
| Cox proportional hazards | Cox-PH |
| Recursively partitioned mixture modelling | RPMM |
| t-distributed random neighbor embedded | t-SNE |
| non-negative matrix factorization | NMF |
| **Evaluation Metrics** |  |
| Area Under the Receiver Operating Characteristic Curve | AUC |
| Matthews Correlation Coefficient | MCR |
| Mean Absolute Residual | MAE |
| Pearson Correlation Coefficient | PCC |
| Root Mean Squared Error | RMSE |
| Concordance index | C-index |

| Table2: List of performance evaluation in cancer epigenetics subtype classification | | | | | | | | | | | | | |
| --- | --- | --- | --- | --- | --- | --- | --- | --- | --- | --- | --- | --- | --- |
| Classification Task (%) | | | | | | | | Regression task | | | | | |
| Ref. | AUC | Accuracy | Precision | Sensitivity | F1 score | Specificity | Matthews correlation coefficient | Root mean square error | Mean absolute residual | R-squared  $R^{2}$ | Correlation coefficient | Pearson correlation coefficient | Spearman correlation coefficient |
| Multi-omics datasets | | | | | | | | | | | | | |
| (Albaradei, Napolitano et al. 2021) | 91.19 | 88.85 | 91.65 | 87.69 | 90.44 | 89.61 | -- | -- | -- | -- | -- | -- | -- |
| (Mallik, Seth et al. 2020) | 85 | 90.69 | 93.38 | 73.97 | -- | 97.63 | -- | -- | -- | -- | -- | -- | -- |
| (Peng, Zheng et al. 2020) | 94.6 | -- | -- | 88.7 | -- | 90 | -- | -- | -- | -- | -- | -- | -- |
| (Seal, Das et al. 2020) | 95.1 | -- | 96 | 95 | 95 | -- |  | 1.33 | -- | 0.96 | 0.68 | -- | -- |
| (Xu, Wu et al. 2019) | -- | 74.32 | 80.66 | 81.33 | 88.66 | -- | -- | -- | -- | -- | -- | -- | -- |
| (Zhang, Xue et al. 2021) | 98 | 98 | 99 | 98 | 99 | -- | -- | -- | -- | -- | -- | -- | -- |
| Unimodal data (DNA methylation) | | | | | | | | | | | | | |
| (Eissa, Khairuddin et al. 2022) | 90.92 | 98.33 | 98.77 | 98.32 | 98.91 | -- | 93.5 | -- | -- | -- | -- | -- | -- |
| (Gomes, Paul et al. 2022) | 98.75 | 98.75 | 99.99 | 97.6 | 98.73 | -- | -- | -- | -- | -- | -- | -- | -- |
| (Jabari, Kobow et al. 2022) | 100 | 94 | 98 | 98 | -- | -- | -- | -- | -- | -- | -- | -- | -- |
| (Levy, Chen et al. 2021) | -- | 98 | 97 | 97 | 97 | -- | -- | -- | -- | -- | -- | -- | -- |
| (Levy, Titus et al. 2020) | -- | 97 | 97 | 97 | 97 | -- | -- | -- | 3.0 | 0.96 | -- | -- | -- |
| (Li, Wei et al. 2021) | 99.69 | -- | -- | 93.94 | -- | 100 | -- | -- | -- | -- | -- | 0.88 | -- |
| (Lin, Hu et al. 2022) | 99.68 | 98.37 | -- | 98.15 | -- | 99.53 | 92.95 | -- | -- | -- | -- | -- | -- |
| (Wang and Wang 2019) | 91.50 | 91.50 | -- | 78.75 | 81.25 | -- | -- | -- | -- | -- | -- | -- | -- |
| (Zheng and Xu 2020) | -- | -- | -- | 94.48 | -- | 99.80 | -- | -- | -- | -- | -- | -- | -- |

| Table3: List of performance evaluation in missing value imputation and prediction of CpG methylation data | | | | | | | | | | | | | |
| --- | --- | --- | --- | --- | --- | --- | --- | --- | --- | --- | --- | --- | --- |
| Classification Task (%) | | | | | | | | Regression task | | | | | |
| Ref. | AUC | Accuracy | Precision | Sensitivity | F1 score | Specificity | Matthews correlation coefficient | Root mean square error | Mean absolute error | R-squared  $R^{2}$ | Correlation coefficient | Pearson correlation coefficient | Spearman correlation coefficient |
| Multi-omics datasets | | | | | | | | | | | | | |
| (Qiu, Zheng et al. 2020) | -- | -- | -- | -- | -- | -- | -- | GE:0.31  DM:0.70 | -- | -- | -- | -- | -- |
| Unimodal data (DNA methylation) | | | | | | | | | | | | | |
| (Angermueller, Lee et al. 2017) | 92.4 | -- | -- | -- | -- | -- | -- | -- | -- | -- | -- | -- | -- |
| (Cheng, Wang et al. 2021) | 96.35 | 93.03 | -- | 86.61 | -- | 93.74 | 63.84 | -- | -- | -- | -- | -- | -- |
| (De Waele, Clauwaert et al. 2022) | 91.68 | -- | -- | -- | -- | -- | -- | -- | -- | -- | -- | -- | -- |
| (Pan, Liu et al. 2019) | -- | -- | -- | -- | -- | -- | -- | -- | 0.03 | -- | -- | 0.82 | -- |
| (Tian, Zou et al. 2019) | 93.2 | 89.9 | -- | 91.3 | -- | 85.3 | -- | 0.21 | 0.19 | -- | -- | -- | -- |
| (Zhou, Chen et al. 2022) | 82.5 | -- | -- | -- | -- | -- | -- | 0.15 | -- | -- | -- | -- | 0.78 |
| Gene Expression Data: GE  DNA Methylation Data: DM | | | | | | | | | | | | | |

| Table 4: List of performance evaluation in identification of epigenetic biomarkers for cancer diagnosis and prognosis | | | | | | | | | | | | | | |
| --- | --- | --- | --- | --- | --- | --- | --- | --- | --- | --- | --- | --- | --- | --- |
| Classification Task (%) | | | | | | | | Regression task | | | | | | |
| Ref. | AUC | Accuracy | Precision | Sensitivity | F1 score | Specificity | Matthews correlation coefficient | Root mean square error | Mean absolute residual | R-squared  $R^{2}$ | Correlation coefficient | Pearson correlation coefficient | Spearman correlation coefficient |  |
| Unimodal data (DNA methylation) | | | | | | | | | | | | | | |
| (Bahado-Singh, Ibrahim et al. 2022) | 100 | -- | -- | 100 | -- | 88 | -- | -- | -- | -- | -- | -- | -- |  |
| (Bahado-Singh, Ibrahim et al. 2022) | 100 | -- | -- | 95 | -- | 100 | -- | -- | -- | -- | -- | -- | -- |  |
| (Liu, Liu et al. 2019) | 98 | 91 | -- | 93.65 | -- | 92.8 | -- | -- | -- | -- | -- | -- | -- |  |

| Table 5: List of performance evaluation in estimating patient survival and survival-sensitive subtypes in cancer | | | | | | | | | | | | |
| --- | --- | --- | --- | --- | --- | --- | --- | --- | --- | --- | --- | --- |
| Classification Task (%) | | | | | | | Survival Task | | | | | |
| Ref. | AUC | Accuracy | Precision | Sensitivity | F1 score | Specificity | | C-index | Log-rank P-value | Pearson correlation coefficient | Brier score |  |
| Multi-omics datasets | | | | | | | | | | | | |
| (Bichindaritz, Liu et al. 2021) | -- | -- | -- | -- | -- | -- | | 0.72 | -- | -- | -- |  |
| (Gu and Zhao 2019) | -- | -- | -- | -- | -- | -- | |  | 4.37e-07 | 0.13 | -- |  |
| (Lee, Huang et al. 2020) | -- | -- | -- | -- | -- | -- | | 0.64 | 9.3e-03 | -- | -- |  |
| (Ma and Zhang 2019) | 78 | -- | 70 | -- | -- | -- | | -- | -- | -- | -- |  |
| (Pan, Burgman et al. 2022) | 99 | 98 | -- | 99 | -- | 96 | | -- | -- | -- | -- |  |
| (Song, Ruan et al. 2022) | -- | -- | -- | -- | -- | -- | | 0.78 | 1.53e-7 | -- | -- |  |
| (Takahashi, Asada et al. 2020) | [43% ,99%] | -- | -- | -- | -- | -- | | -- | 0.003 | -- | -- |  |
| (Tian, Zhu et al. 2022) | -- | -- | -- | -- | -- | -- | | 0.92 | 0.0001 | -- | 0.16 |  |
| (Tong, Mitchel et al. 2020) | -- | -- | -- | -- | -- | -- | | ConcateAE: 0.64  CrossAE: 0.63 | -- | -- |  |  |
| Unimodal data (DNA methylation) | | | | | | | | | | | | |
| (Massi, Dominoni et al. 2022) | -- | -- | -- | -- | -- | -- | | 0.70 | -- | -- | -- |  |

| Table 6: List of performance evaluation appearing in this paper | |
| --- | --- |
| Performance matric | Criterion |
| Sensitivity (Recall) | $\frac{TP}{TP+FN}$ |
| Specificity | $\frac{TN}{TN+FP}$ |
| Accuracy | $\frac{TP+TN}{TP+TN+FP+FN}$ |
| Matthews correlation coefficient | $\frac{TP\times TN-FP\times FN}{\sqrt{(TP+FP)\times(TP+FN)\times(TN+FP)\times(TN+FN)}}$ |
| Precision | $\frac{TP}{TP+FP}$ |
| F1-score | $\frac{2\times Precision\times Recall}{\mathrm{Precision}+\mathrm{Recall}}$ |
| Root mean square | $\sqrt{\frac{1}{N}\sum_{i=1}^{N} {{(y}_{i}-\hat{y_{i}})}^{2}}$ |
| Mean absolute error | $\frac{1}{N}\times\sum_{1}^{N} \left\vert{(y}_{i}-\hat{y_{i}} \right\vert$ |
| R-squared ($\boldsymbol{R}^{\mathbf{2}}\boldsymbol{)}$ | $R^{2}=1-(\frac{SSres}{SStot})$  $SSres=\sum{{(y}_{i}-\hat{y_{i}})}^{2}$  $SStot=\sum{{(y}_{i}-\bar{y})}^{2}$ |
| Correlation coefficient | $\frac{Cov(y_{i},\hat{y_{i}})}{\sigma_{y_{i}}\times\sigma_{\hat{y_{i}}}}$ |
| Pearson correlation coefficient | $\frac{Cov(y_{i},\hat{y_{i}})}{\sigma_{y_{i}}\times\sigma_{\hat{y_{i}}}}$ |
| Spearman correlation coefficient | $\frac{Cov({(y}_{i},\hat{y_{i}})}{\sigma_{{rank(y}_{i)}}\times\sigma_{rank(\hat{y_{i)}}}}$ |
| C-index | $pr\{\left. H_{i}>H_{j} \right\vert T_{i}<T_{j} ,C_{i}=1 \}$ |
| Definition:  Correlation Coefficient: the correlation coefficient measures the linear relationship between calculated and observed values.  Pearson correlation coefficient: the Pearson correlation coefficient measures the linear relationship between two standardized calculated and observed values.  Spearman correlation coefficient: The Spearman correlation coefficient measures the non-linear relationships between the ranks of calculated and observed values.  C-index: It is calculated by comparing the predicted risks of the model to the actual outcomes of the events (Uno, Cai et al. 2011).  Log- rank $\boldsymbol{p}$ value: the survival difference between subgroups (Lin and Zelterman 2002).  Brier score: the accuracy of probabilistic prediction (Rufibach 2010). | |
| Parameters:  *TN*= the numbers of the true negative,  *TP*= the numbers of true positive,  *FN*= the numbers of false negative,  *FP*= the number of false positive  *N*= the number of the data samples,  $\boldsymbol{y}_{\boldsymbol{i}}$=the actual value of the target variable for the i-th sample  $\hat{\boldsymbol{y}_{\boldsymbol{i}}}$=the predicted value of the target variable for the i-th sample  C*ov* = the covariance of the calculated and observed values  $\boldsymbol{\sigma}$= the standard deviation  $\bar{\boldsymbol{y}}$= the mean value of the target variable over all samples in the dataset  $\boldsymbol{SSres}$= The sum of squared residuals  $\boldsymbol{SStot}$= the total sum of squares  Rank: the ranks of the calculated and observed values  $\boldsymbol{H}_{\boldsymbol{i}}$= the hazard for the i-th patient  $\boldsymbol{T}_{\boldsymbol{i}}$= the survival time for the i-th patient | |
